# Comparative transcriptome analysis reveals potentially novel roles of Homeobox genes in adipose deposition in fat-tailed sheep

**DOI:** 10.1038/s41598-017-14967-9

**Published:** 2017-11-03

**Authors:** Danju Kang, Guangxian Zhou, Shiwei Zhou, Jie Zeng, Xiaolong Wang, Yu Jiang, Yuxin Yang, Yulin Chen

**Affiliations:** College of Animal Science and Technology, Northwest A&F University, No. 22, Xinong Road, Yangling, 712100 Shaanxi China

## Abstract

Adipose tissues are phenotypically, metabolically and functionally heterogeneous based on the sites of their deposition. Undesirable fat deposits in the body are often detrimental to animal and human health. To unravel the potential underlying mechanisms governing accumulation of adipose tissues in various regions of the body, i.e., subcutaneous (SAT), visceral (VAT) and tail (TAT), we profiled transcriptomes from Tan sheep, a Chinese indigenous breed with notable fat tail using RNA-seq. Upon comparison, we identified a total of 1,058 differentially expressed genes (DEGs) between the three adipose types (218, 324, and 795 in SAT/VAT, SAT/TAT, and VAT/TAT, respectively), from which several known key players were identified that are involved in lipid metabolic process, Wnt signals, Vitamin A metabolism, and transcriptional regulation of adipocyte differentiation. We also found that many elevated genes in VAT were notably enriched for key biological processes such as cytokine secretion, signaling molecule interaction and immune systems. Several developmental genes including HOXC11, HOXC12 and HOXC13, and adipose-expressed genes in the tail region, such as HOTAIR_2, HOTAIR_3 and SP9 were specially highlighted, indicating their strong associations with tail fat development in fat-tailed sheep. Our results provide new insight into exploring the specific fat deposition in tail, also contribute to the understanding of differences between adipose depots.

## Introduction

Adipose tissue is a heterogeneous mini-organ composed primarily of adipocytes that are surrounded by fibroblasts, fibroblastic preadipocytic cells, endothelial cells, nerves and immune cells^1^. It has an enormous expansion capacity by increasing adipocyte number and size, potentially leading to obesity. Excessive fat accumulation may lead to metabolic consequence, such as cardiovascular disease, hypertension and type 2 diabetes, which are related more to body fat distribution than total body fat^2^. In adult mammals, there are two main adipose types based on their anatomical locations, subcutaneous and visceral adipose tissues. Subcutaneous adipose tissue (SAT) broadly lies below the dermis, and functions as the main energy storage area, while visceral adipose tissue (VAT) is wrapped around the internal organs, and provides protective padding. Cells of the two types of fat tissues are recognized to be morphologically, metabolically and functionally distinct^3^. The VAT adipocytes are more metabolically active, more sensitive to lipolysis, possess a lesser potential for accumulating lipid and have a greater percentage of large adipocytes when comparted with SAT in pigs^2^. VAT has a greater capacity to generate free fatty acids (FFAs) while SAT is more active in absorption of circulating FFAs and triglycerides^2^.

While much is known about SAT and VAT, tailed adipose tissue (TAT) on the other hand does not seem to receive considerable attention. Currently, to the best of our knowledge, very few animals such as sheep possess the ability to store so large amounts of fat in the tail region. For example, in Barbarine, an extremely fat-tailed sheep breed in Tunisia, the weight of lamb tail fat can reach up to 50% of the total body fat and more than 10% of the carcass^4^. These fat-tailed sheep reportedly accounted for a significant percentage of the world sheep population^5^. Their tail fats are considered to be important sources of energy, especially for adult ewes. During adverse conditions, such as upon food scarcity resulting from migration, drought and winter, this body reserve from the fat tail is actively mobilized to provide energy as a survival buffer, accompanied by an enormous change in tail weight, without dramatically altering the physiology of the animal^6^. It is also noteworthy that tail fat was once considered a desirable food among local residents to resist the cold. However, the very large fat tail not only resulted in immensely reduced carcass quality, but also tedious for the shepherds, as they had to provide necessary assistance at mating time. Furthermore, the eating habits of consumers have changed largely in recent years, with lesser preference for fatty meat.

Tan sheep with a medium sized fat tail are largely distributed in the arid regions of Ningxia Province in northwestern China. A few recent advances have successfully employed RNA-seq techniques to survey gene expression profiling in various fat depots in humans, pigs and cattle^3,7–9^. There have also been a few comparative transcriptome studies between different sheep breeds^10,11^. Two genomic regions located on Chromosomes 5 and X were proposed to be strongly correlated with tail fat deposition in fat-tailed sheep^12^. Yuan *et al*.^13^ have screened a group of genes that are potentially associated with tail fat development through genome selection signature analysis. However, we think that more complex gene networks are linked to tail fat accumulation. Genetic determinants responsible for such phenotypic, distributional, and functional differences among various fat compartments, particularly in adipose tissues from fat tails, are still elusive. In this study, we discovered transcriptomic differences among subcutaneous, visceral and tail adipose tissues in a fat-tailed sheep breed, thereby facilitating elucidation of the molecular mechanisms of fat distribution and accumulation among different adipose depots. Our study lays the foundation for addressing this issue in future studies of sheep tail fat and enhancing our understanding of human regional obesity.

## Results

### Differences in adipocyte phenotype and fatty acid composition

**Figure 1:**
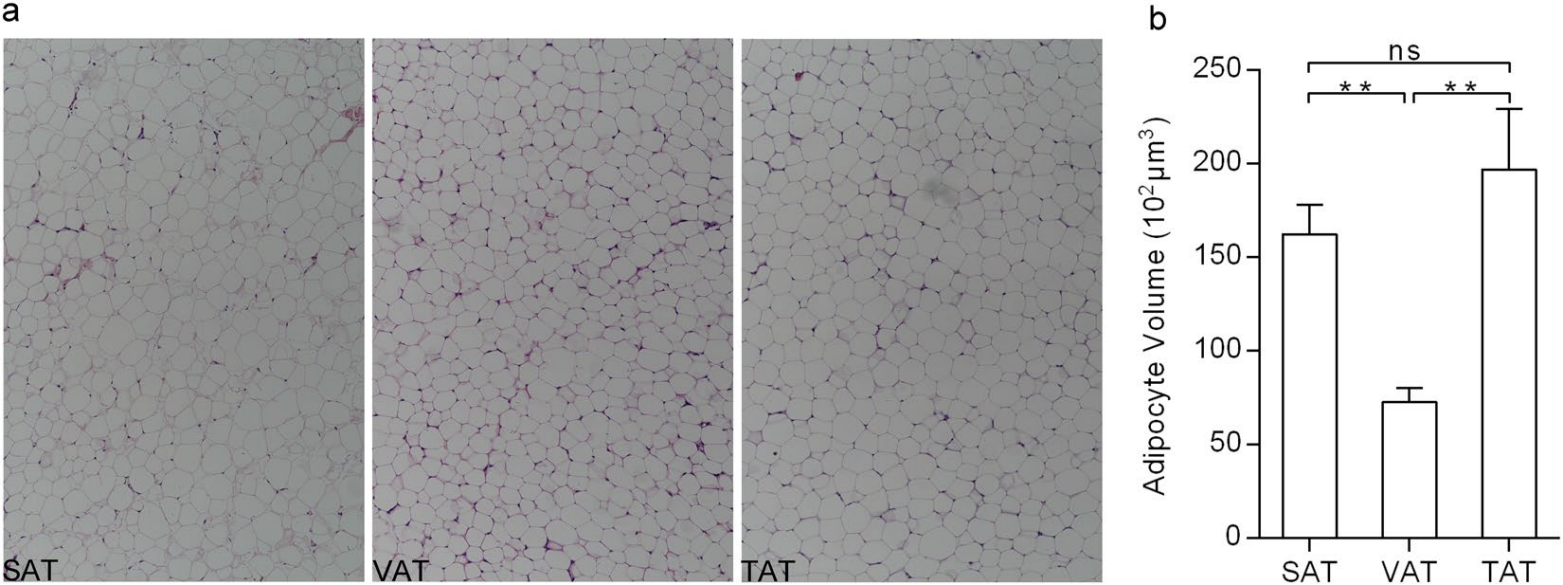
Phenotype analysis of the three adipose depots. (**a**) Adipocytes were photographed at 200 × magnification. (**b**) Adipocyte diameter of SATs, VATs and TATs. One-way ANOVA and Tukey multiple range test, values were expressed as mean ± SE, * represents *p* < 0.05, ns: not significant.

**Figure 2:**
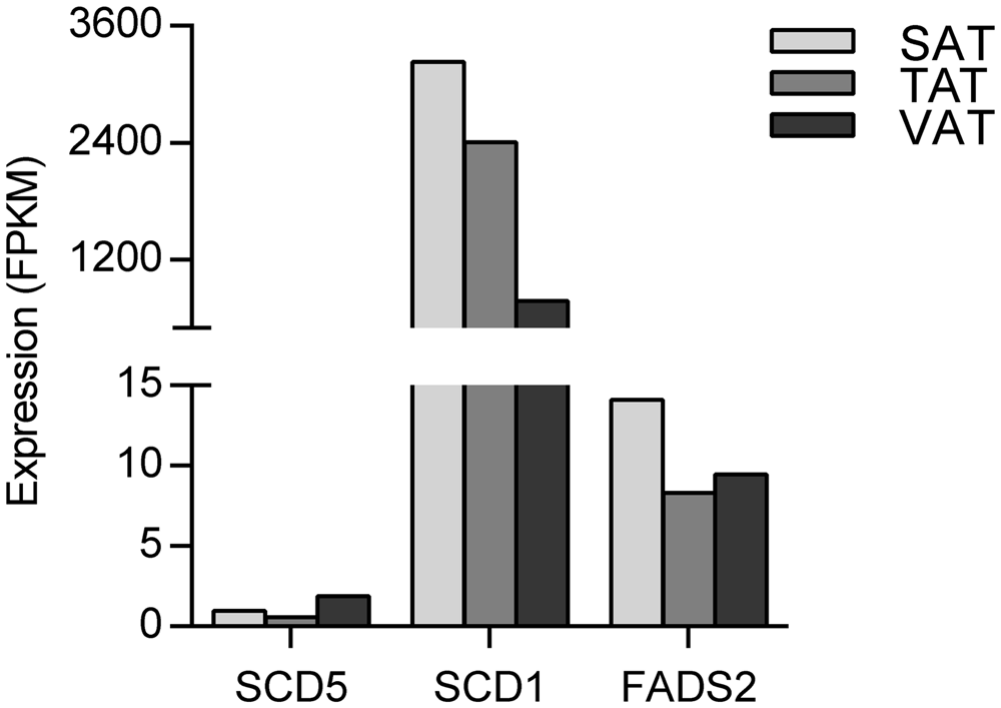
Changes in expression of genes that influence on fatty acid composition. SCD1, SCD5 and FADS2 were found to be involved in fatty acid metabolism in SAT, TAT and VAT. Y-axis represents the expression levels of three genes; the expression value is denoted with FPKM from RNA-seq data.

We found TAT and VAT exhibited marked variations in adipocyte diameter, while small differences were observed between TAT and SAT, not reaching the significant change (Fig. 1a and b), may imply greater intrinsic differences in TAT vs. VAT than in TAT vs. SAT. Table 1 presented variations in fatty acid composition among the three different anatomical fat regions. Data showed that TAT and SAT had the tendency of containing higher monounsaturated fatty acids (MUFA) but lower saturated fatty acids (SFA) than VAT, which may be reflected by the higher expression of SCD1 (a key desaturase, converts saturated C16:0 and C18:0 to their monounsaturated counterparts) in TAT and SAT but lower in VAT (Fig. 2), however, there was no significant difference between SAT and VAT, indicating that major fatty acids in the tail adipose tissue were significantly different with the other two adipose tissues, subcutaneous and visceral adipose tissues. An isoform of the SCD family, SCD5 was a preferentially expressed in VAT (Fig. 2), suggesting that SCD1 and SCD5 play distinct roles in fatty acid metabolism. In addition, higher expression of FADS2 in SAT than in VAT, was consistent with C18:2n-6 (SAT: 0.115 ± 0.025%, VAT: 0.181 ± 0.037%) and C18:3n-6 (SAT: 2.430 ± 0.400%, VAT: 2.316 ± 0.107%) contents, because the enzymatic activity of Δ-6 fatty acid desaturase FADS2 is responsible for the conversion of 18:2(n-6) to 18:3(n-6) (Table 1, Fig. 2).

**Table 1:** Difference in fatty acid composition from three adipose sites.

Fatty acid (g·100 g^−1^)	SAT	VAT	TAT	*P*	*P-*Value		
					*P1*	*P2*	*P3*
C14:0 (Myristic)	2.981 ± 0.114	3.401 ± 0.285	2.413 ± 0.201	0.044	0.397	0.220	0.038
C16:0 (Palmitic)	24.314 ± 1.124	28.494 ± 1.635	23.402 ± 0.799	0.056	0.117	0.864	0.060
C18:0 (Stearic)	28.005 ± 1.094	38.885 ± 1.344	22.224 ± 1.465	0.000	0.003	0.047	0.000
SFA	59.901 ± 1.758	66.180 ± 2.255	48.039 ± 2.247	0.002	0.167	0.017	0.002
C16:1n-7 (Palmitoleic)	2.002 ± 0.102	1.871 ± 0.172	2.690 ± 0.189	0.022	0.833	0.051	0.025
C18:1n-9 (Oleic)	35.013 ± 1.936	29.025 ± 2.174	46.326 ± 1.962	0.003	0.172	0.018	0.002
MUFA	37.015 ± 2.030	30.896 ± 2.319	49.015 ± 2.073	0.003	0.189	0.018	0.002
C18:2n-6 (Linoleic)	0.115 ± 0.025	0.181 ± 0.037	0.172 ± 0.032	0.347	0.369	0.461	0.978
C18:3n-6 (γ-linolenic)	2.430 ± 0.400	2.316 ± 0.107	2.251 ± 0.208	0.893	0.951	0.885	0.984
C18:3n-3 (α-linolenic)	0.337 ± 0.047	0.376 ± 0.060	0.403 ± 0.082	0.780	0.906	0.764	0.956
C20:4n-6 (ARA)	0.137 ± 0.014	0.047 ± 0.024	0.062 ± 0.013	0.024	0.027	0.056	0.817
C20:5n-3 (EPA)	0.025 ± 0.007	0.004 ± 0.004	0.033 ± 0.000	0.011	0.039	0.503	0.010
C22:6n-3 (DHA)	0.040 ± 0.024	0.000 ± 0.000	0.026 ± 0.004	0.207	0.191	0.764	0.449
PUFA	3.084 ± 0.444	2.923 ± 0.198	2.946 ± 0.273	0.930	0.934	0.951	0.999

### Summary of transcriptome sequencing data

We sequenced 9 cDNA libraries from three adipose depots from Tan sheep (three replicates each of SAT, VAT and TAT depots), and obtained 61,512,242–80,602,654 paired-end fragments of 150 bp in length. The detailed classification of the raw reads can be found in the supplementary material (Fig. S1). A total of 93.12 Gb clean bases were generated after rejecting low quality reads, which were then aligned against the sheep genome Oar_v3.1. The basic alignment information is shown in Table 2. Of the total matched sequences, approximately 70.42–76.36% sequences had a unique genomic location in three types of adipose tissue, from among which 67.6–76.2% of the sequences were assigned to annotated exons and the remaining reads were assigned to intergenic and intron regions (Fig. S2).

**Table 2:** The basic alignment information for RNA-seq data generated from three adipose depots of the fat-tailed Tan sheep.

Mapping summary	TS1			TS2			TS3		
	SAT1	VAT1	TAT1	SAT2	VAT2	TAT2	SAT3	VAT3	TAT3
Raw reads	65,880,104	80,602,654	61,512,242	78,707,478	69,633,124	74,442,636	67,114,874	71,803,958	78,599,898
Clean reads	64,178,150	76,739,802	58,169,446	76,268,478	66,696,618	70,949,286	64,979,520	68,344,744	74,468,172
Clean reads: Q20	97.68%	97.09%	96.81%	97.43%	97.15%	97.03%	97.39%	97.16%	97.06%
Clean reads: Q30	94.25%	93.00%	92.49%	93.87%	93.05%	92.89%	93.74%	93.12%	92.98%
Clean reads: GC content	50.83%	54.12%	53.04%	51.25%	51.52%	54.49%	53.27%	54.04%	53.26%
Clean reads: error rate	0.01%	0.01%	0.01%	0.01%	0.01%	0.01%	0.01%	0.01%	0.01%
Total mapped reads	50,796,443	56,521,253	42,399,979	59,273,330	51,589,040	52,650,369	49,932,739	50,596,334	55,356,714
Multiple mapped reads	1,791,049	2,132,453	1,436,691	1,913,134	1,906,062	1,657,038	1,624,543	2,053,102	2,086,288
Unique mapped reads	49,005,394	54,388,800	40,963,288	57,360,196	49,682,978	50,993,331	48,308,196	48,543,232	53,270,426
Non-splice reads	28,450,457	32,733,131	25,511,843	34,017,881	29,584,890	28,299,573	25,897,178	28,482,876	33,067,759
Splice reads	20,554,937	21,655,669	15,451,445	23,342,315	20,098,088	22,693,758	22,411,018	20,060,356	20,202,667
Mapping rate	79.15%	73.65%	72.89%	77.72%	77.35%	74.21%	76.84%	74.03%	74.34%

### Differentially expressed genes in SAT, VAT, and TAT

Firstly, we estimated gene expression levels for each sample by using HTSeq software. As shown in Table S1, around 6% of 29,644 annotated genes were expressed more than 60 FPKM; about 11% were expressed between 15–60 FPKM, 18% between 3–15 FPKM, and 10% between 1–3 FPKM. The rest were expressed less than 1 FPKM. To ensure the reliability of the results for further analysis, pair-wise correlation between any two biological replicates in each group was checked based on the normalized FPKM values. The correlation coefficients were no less than 0.95 (Fig. S3), suggesting a high level of reproducibility and rationality of sample selection.

**Figure 3:**
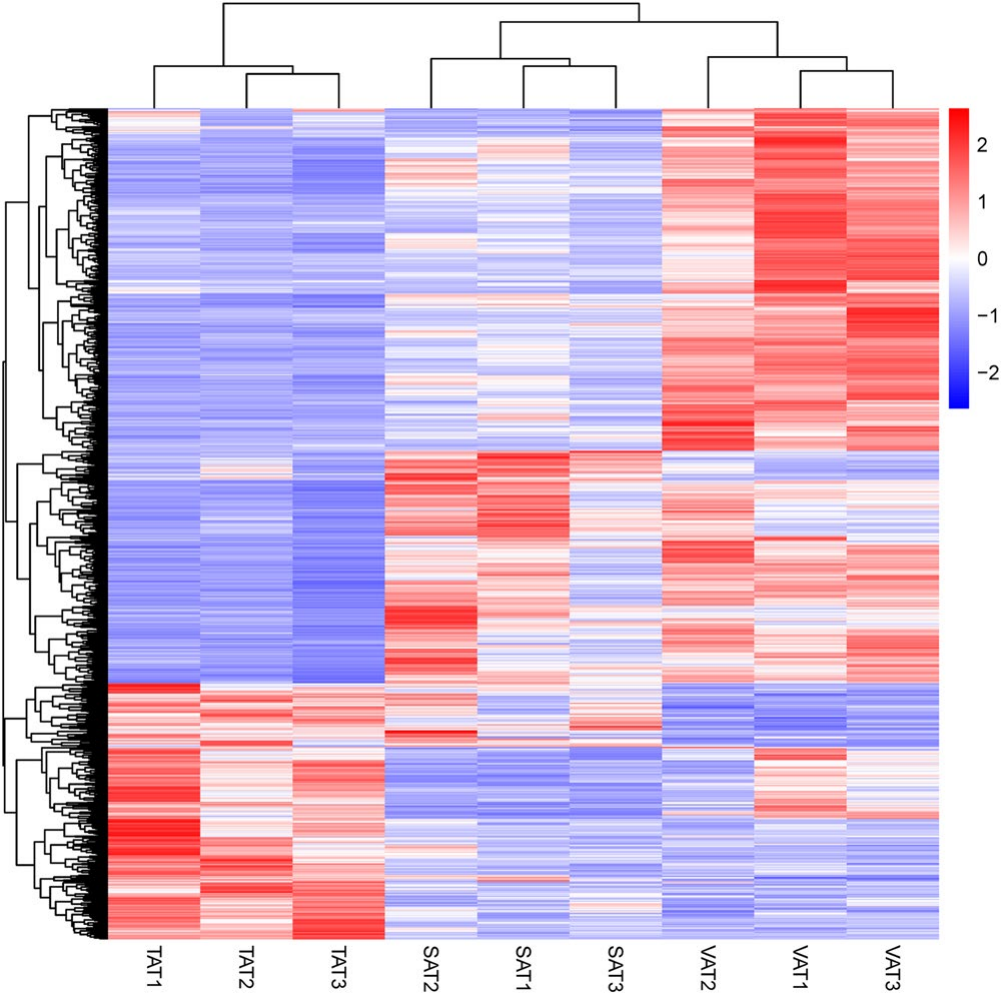
Clustering analysis of 1,058 DEGs in three adipose depots. Genes and samples were clustered in columns and rows using the MultiExperiment Viewer (MeV 4.9.0). The dendrogram was created to intuitively reflect the global expression patterns of DEGs in nine adipose libraries. All libraries are orderly labeled with SAT2, SAT1, SAT3, VAT3, VAT1, VAT2, TAT3, TAT1, and TAT2 at the bottom of each column. Up- and down-regulation are separately colored in red and green.

**Figure 4:**
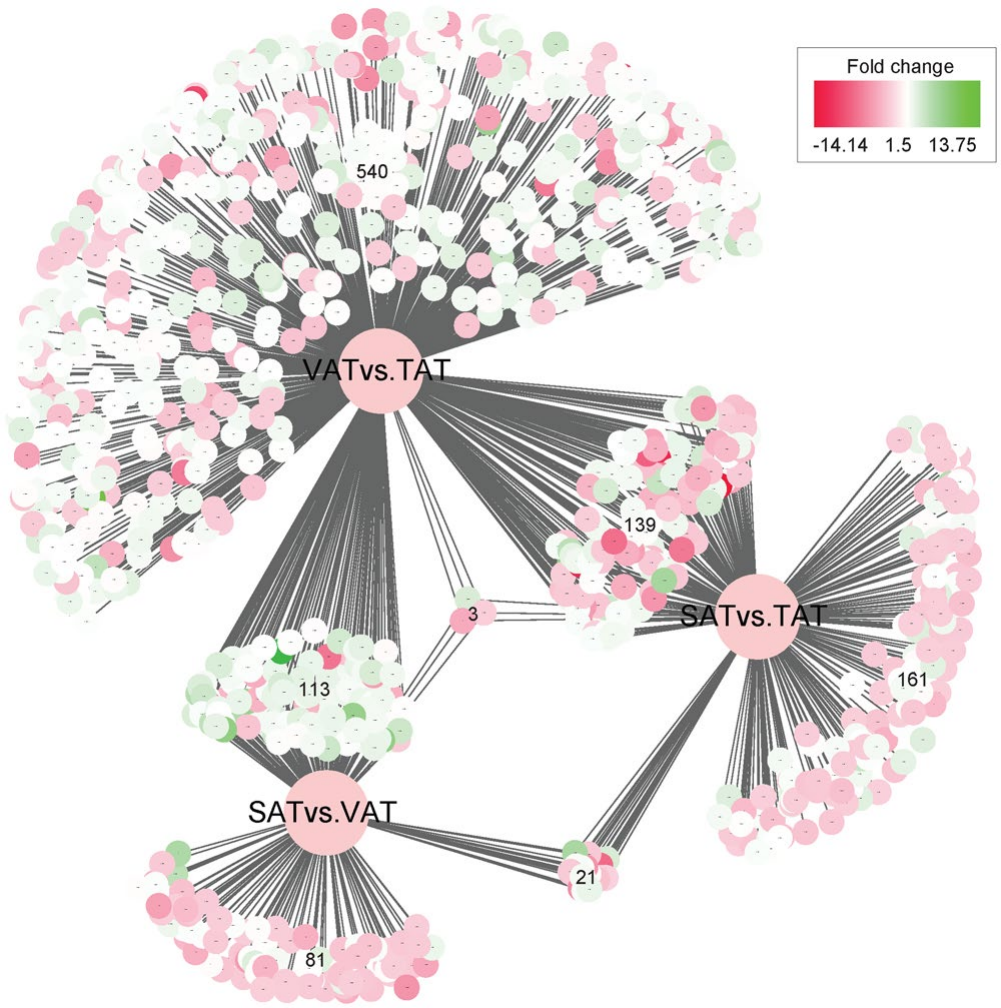
The distribution of DEGs and their fold changes between three adipose depots. A total of 1,058 DEGs were identified in SAT vs. VAT, SAT vs. TAT, and VAT vs. TAT. A large pool of 795 genes was exclusively expressed in the VAT vs. TAT, while a smaller proportion of 218 and 324 genes was expressed uniquely in the SAT vs. VAT and SAT vs. TAT respectively. Three genes shared in all three tissues. Color-coding denotes fold change between any two of the three adipose depots.

**Figure 5:**
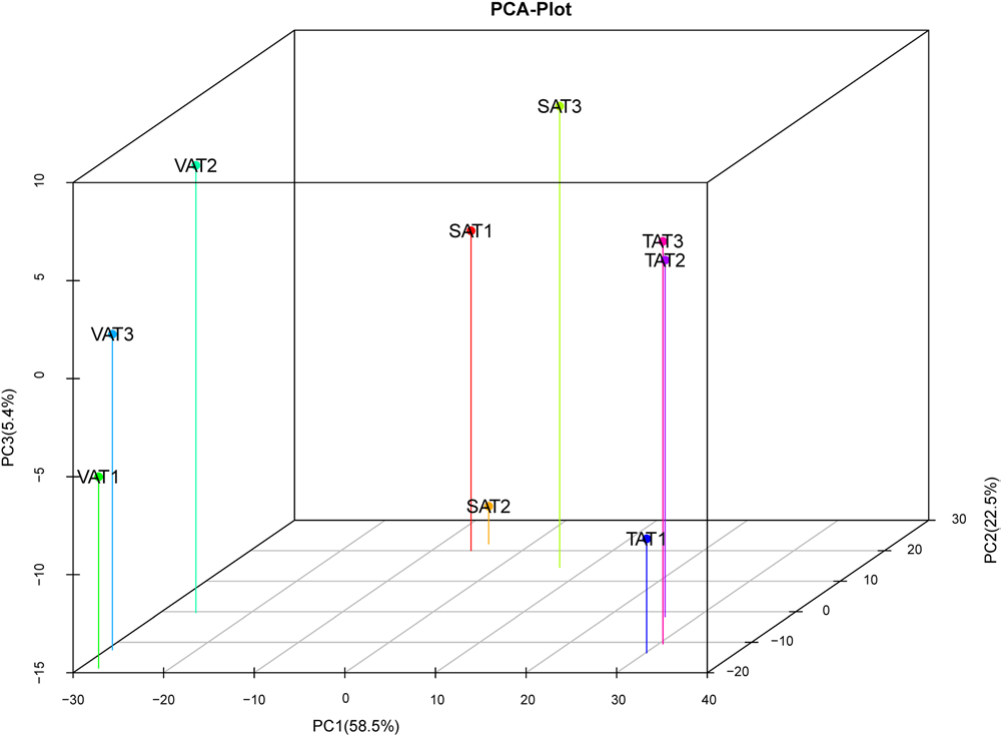
3D PCA plot for the expression profile of genes in the sheep adipose tissue. The PCA plot illustrates the principal components of all nine fat samples from three surveyed tissues. Different individual fat samples from the same adipose type grouped together, and three different adipose types were clearly separated from each other.

To better investigate the differences in gene expression patterns among adipose tissues of SAT, VAT, and TAT, we used a t-test (|log_2_^Ratio^| ≥ 1, adjusted *p*-value ≤ 0.05) to identify differentially expressed genes (DEGs) between any two adipose depots (SAT vs. VAT, SAT vs. TAT, and VAT vs. TAT) (Table S2). A total of 1,058 DEGs were identified in SAT vs. VAT, SAT vs. TAT, and VAT vs. TAT, and were clustered by a visual heat map (Fig. 3). Among these, the numbers of DEGs in SAT/VAT, SAT/TAT and VAT/TAT were 218 (49 up-regulated in SAT and 169 in VAT), 324 (165 up-regulated in SAT and 159 in TAT) and 795 (571 up- regulated in VAT and 224 in TAT) respectively (Fig. 4 and Fig. S4). A relatively higher difference between VAT and TAT, in contrast to SAT and TAT or SAT and VAT indicated that tail fat deposition was distinct from visceral fat storage at the transcriptional level. To further test the relationships among depots, we carried out principal components analysis (PCA) using the FPKM values of these genes from the three adipose types (Fig. 5). The 3D plot displayed the first and second (PC1 and PC2) principal components, with variances of 58.5% and 22.5%, respectively. We observed that the different data sets from the same adipose types grouped together and three different types of adipose tissues were clearly separated from each other. TATs are distinguished from SATs and VATs, and TATs are much closer to SATs than VATs as expected, which further strengthened our assumption that TAT vs. VAT had greater differences than TAT vs. SAT at the molecular level.

**Figure 6:**
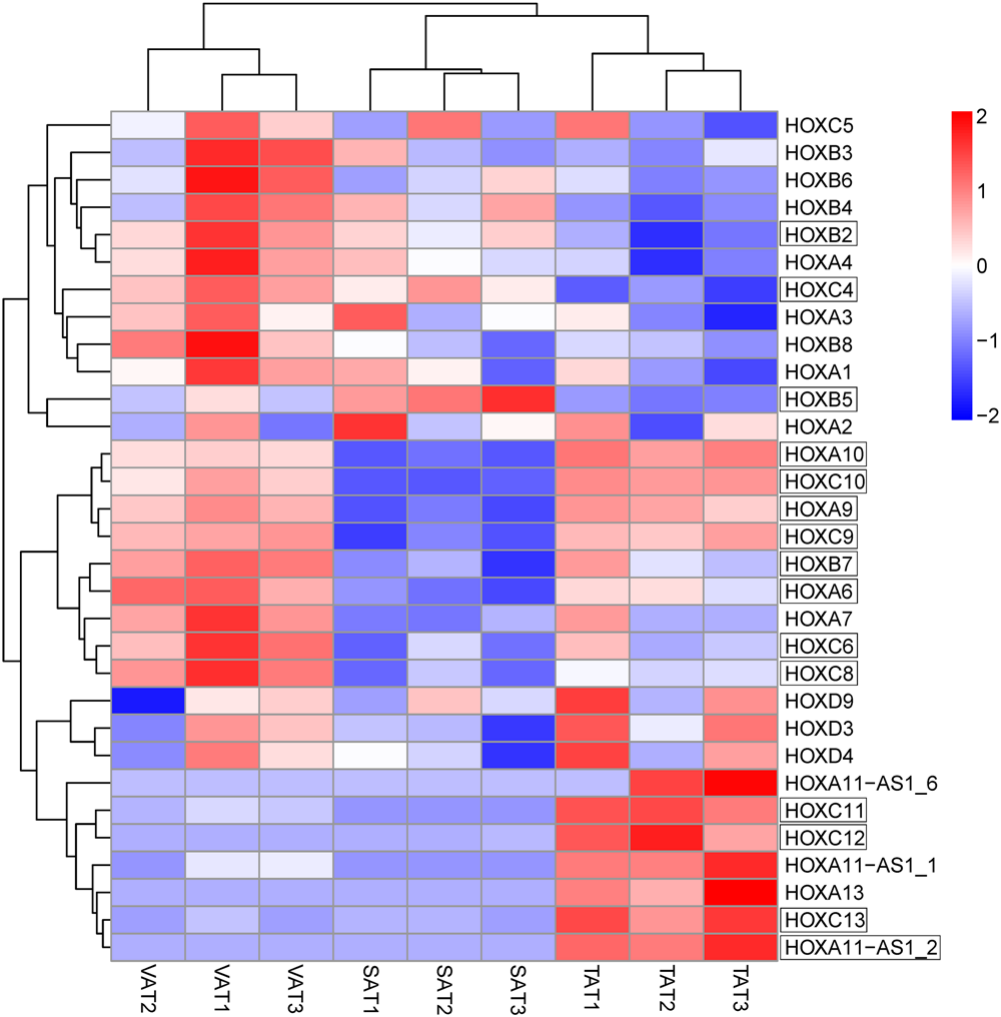
Expression pattern of 31 HOX genes in the three adipose depots was represented in the heatmap. Of these, 15 DEGs were highlighted with rectangular boxes.

**Figure 7:**
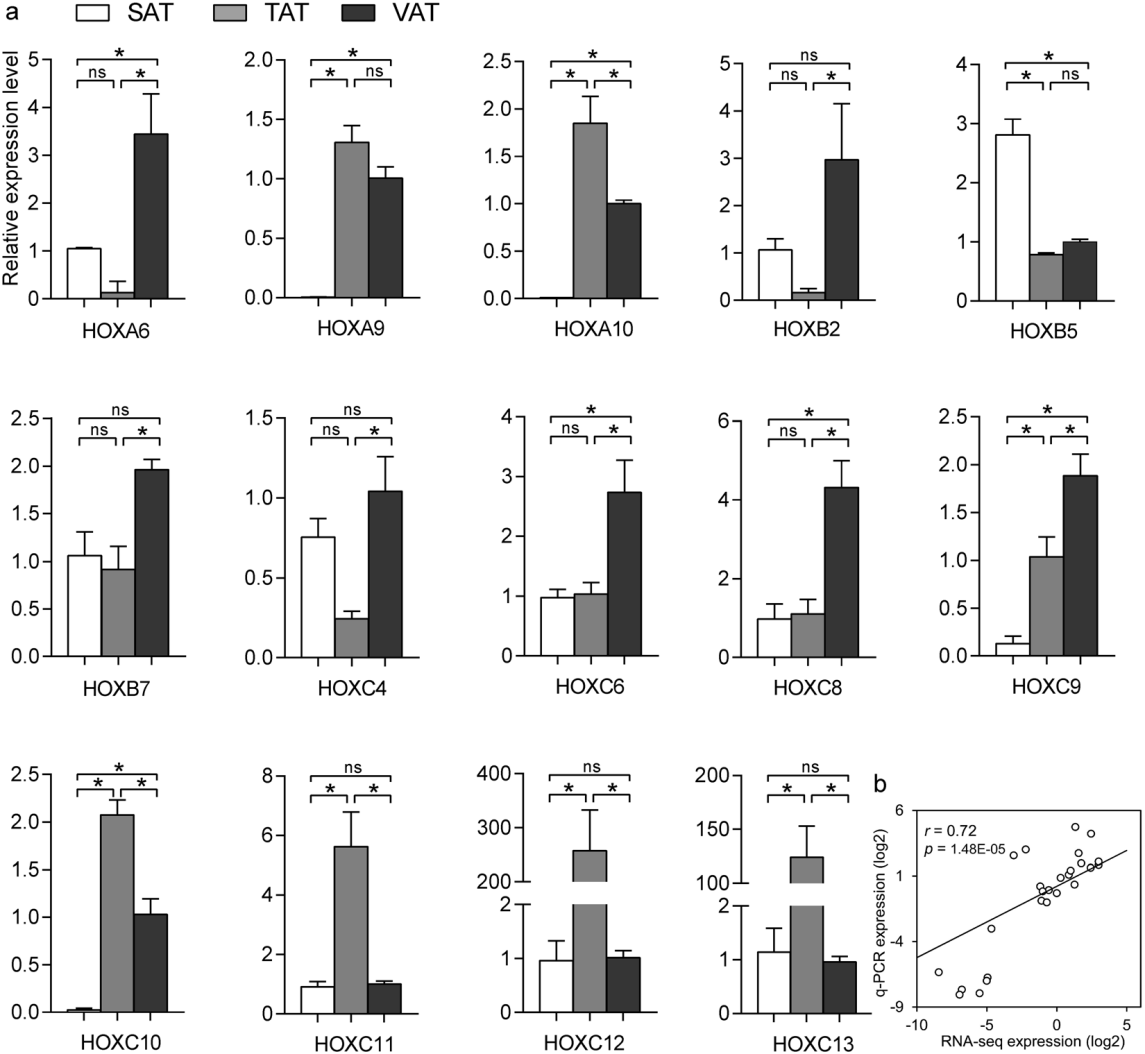
HOX gene expression patterns in three adipose tissues. (**a**) Fourteen differentially expressed HOX genes were measured using q-PCR. Data are expressed as mean ± SE. Statistical significance was examined using One-way ANOVA and the Tukey multiple range test, * indicates *p* < 0.05, ns: not significant. (**b**) Correlation between gene expressions as measured using q-PCR and RNA-seq methods.

Notably, our analysis observed that a specific set of homeobox family proteins encoding genes displayed marked differences in the three fat depots. These gene clusters were known to be implicated in animal development, and have been demonstrated to regulate transcription during human and 3T3-L1 adipogenesis^14,15^. At least 39 homeobox members in humans and mice are located on four different chromosomes. If a gene met the criterion of FPKM ≥ 1 in a sample, it was considered to be expressed in the sample^16^. We found that 28 out of 39 HOX genes in sheep (Oar_v4.0) were expressed in different adipose depots, of which 14 were DEGs; 3 HOXA11- antisense RNA genes were also expressed (Fig. 6). Of these DEGs, the locus A HOX genes on chromosome 4 were mostly active in the visceral and tail fat areas. The locus B genes on chromosome 11 showed a heterogeneous expression in three depots. Several locus C genes including HOXC4, HOXC6, HOXC8 and HOXC9 on chromosome 3 were primarily expressed in the visceral fat, while HOXC10, HOXC11, HOXC12 and HOXC13 were expressed at low or undetectable levels in the subcutaneous depot but high in tail fat (Fig. 6). To confirm expression differences of these genes between multiple regional adipose depots, we measured the 14 HOX genes using real-time quantitative PCR (q-PCR) and found that the results were in agreement with our sequencing data (Fig. 7a and b). Moreover, we compared the detected HOX genes in Tan sheep with those in goats, cow, mice and humans based on homeodomain sequence similarities. The five species and their corresponding HOX genes on chromosomes are summarized in Table S3. Using publicly available data in a published research^9^, we compared expression of the HOX genes in the intramuscular, subcutaneous, and omental adipose tissues of cow and found that 17 HOX genes were differentially expressed between the three white adipose depots, exhibiting distinct differences in expression levels. We also identified 3 HOX-related genes belonging to non-protein coding genes. HOTAIR_2 and HOTAIR_3 positioned within the HOXC cluster, were exclusively expressed in tail fat but almost no expression was observed in other two compartments while HOTAIRM1_4 was expressed with a relatively lower abundance in TAT than in SAT or VAT (Table S2). These results appeared to be consistent with previous reports that lncRNA HOTAIR may modulate key processes during adipocyte differentiation^17^.

### Validation of RNA-seq results by q-PCR

**Figure 8:**
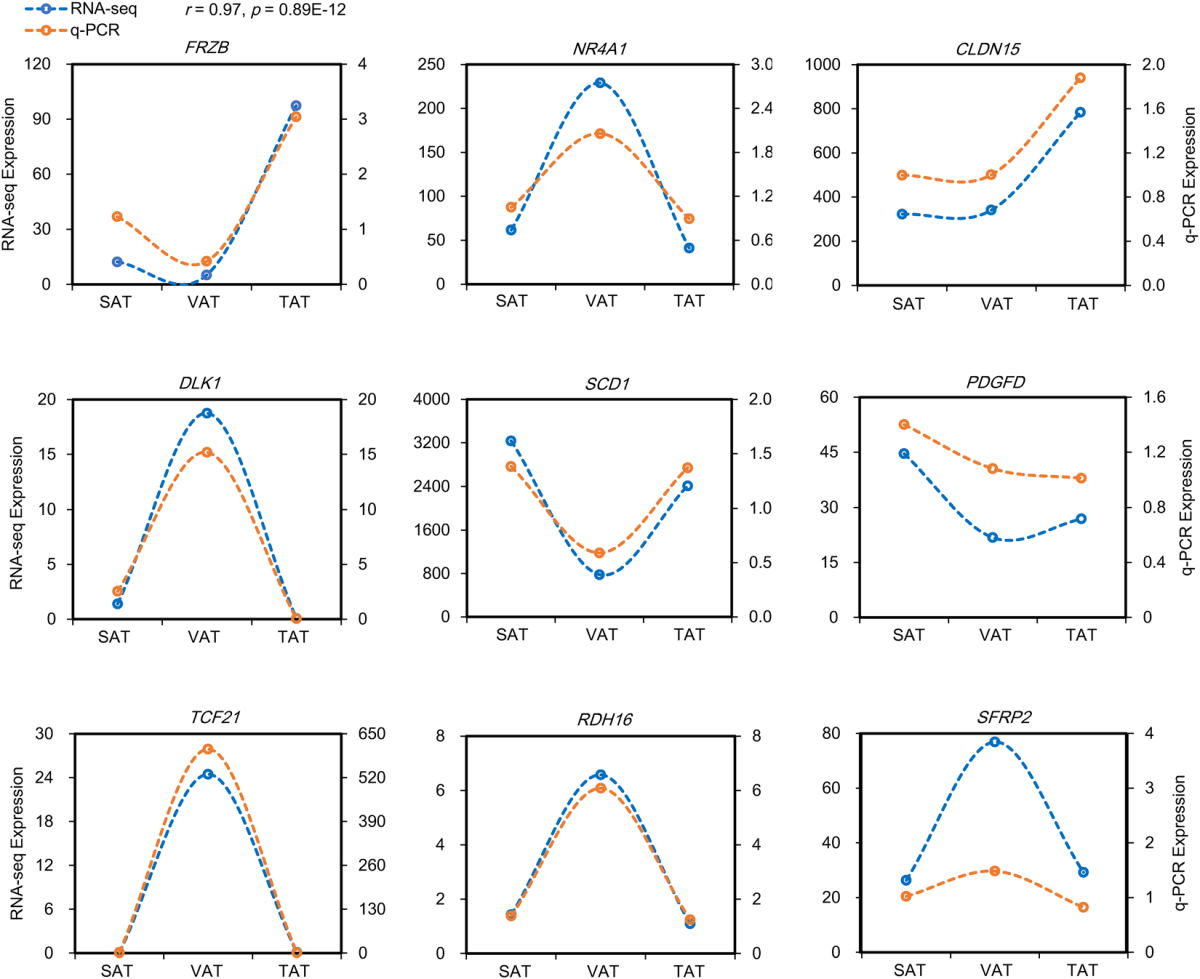
Validation of gene expression by q-PCR. The data presented in Y-axis indicate mRNA expression as determined by q-PCR and RNA-seq. The Pearson correlation coefficient (*r*) and the *p*-value are shown for the nine genes.

To verify the credibility and dependability of the transcriptome data, we randomly chose 9 genes from SAT, VAT and TAT libraries to quantify their expression changes by q-PCR. Most of the genes showed almost identical expression patterns to RNA-seq in all three fat depots (Fig. 8). Linear regression analysis of all these gene expression ratios exhibited a remarkably high correlation (*r* = 0.97, *p* = 8.09E-12; Fig. S5) between the RNA-seq and q-PCR methods, thus increasing confidence in the results obtained from RNA-sequencing technology.

### Gene Ontology enrichment analysis of the DEGs

**Figure 9:**
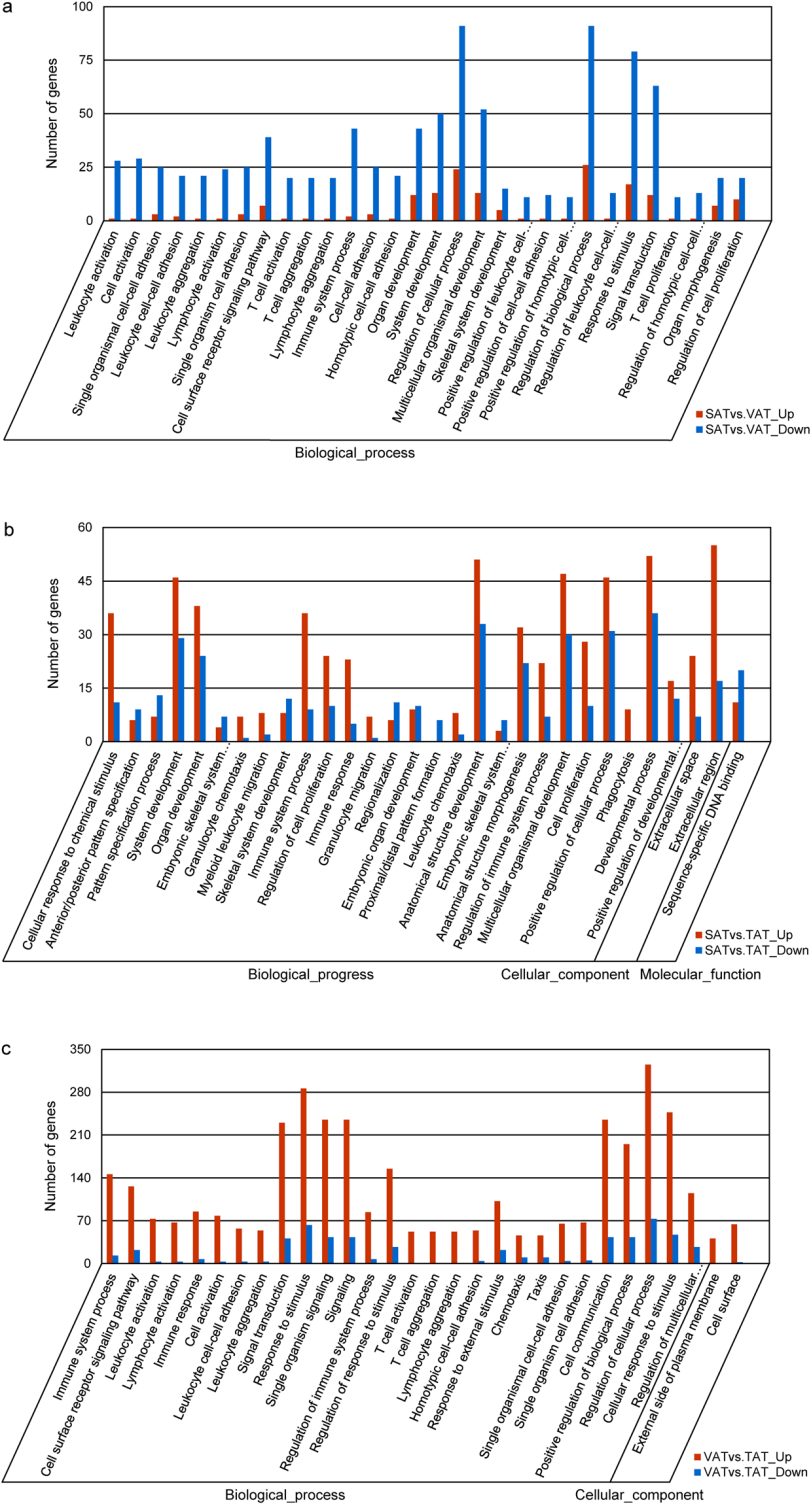
The most enriched 30 GO functional categories for DEGs from three groups of pairwise comparisons. The y-axis presents the number of DEGs in a category. The x-axis shows the specific GO term. Red color bars denote up-regulation and blue color bars denote down-regulation.

To gain valuable insight into the molecular functions of the genes potentially associated with three adipose depots, we categorized the 1,058 identified DEGs into three functional groups depending on gene ontology: biological process, cellular component, and molecular function based on their sequence homologies. Among these three pairwise comparisons, the five most abundant GO terms with significant enrichment (corrected *p*-value ≤ 0.05) were biological process, cellular process, single-organism process, single-organism cellular process, and biological regulation for biological process category (Table S4); membrane, membrane part, extracellular region, cell periphery, and extracellular region part for cellular component category (Table S5); binding, protein binding, molecular transducer activity, receptor activity, and signal transducer activity for molecular function category (Table S5). The number of corresponding genes in each term is listed in Table S4 and Table S5. Leukocyte activation (29 genes, *q* = 4.98E-11), cellular response to chemical stimulus (47 genes, *q* = 5.43E-08), and immune system process (159 genes, *q* = 2.84E-37) were the most prominently enriched GO terms in SAT/VAT, SAT/TAT, VAT/TAT, respectively. A large number of DEGs between SAT and VAT, and between VAT and TAT were found to be involved in inflammation and immune-related response, which explained the presence of distinct inflammatory characteristics between visceral and the other two subcutaneous fat tissues. The top 30 enriched GO functional groups for the DEGs from three comparison groups have been separately shown in Fig. 9a,b and c.

### KEGG pathway analysis of the DEGs

KEGG enrichment analysis of DEGs resulting from the three groups of pairwise comparisons was conducted, indicating that 42.0% (444/1,058) of DEGs were involved in 237 pathways. Among these pathways, “Metabolic pathways” (46 DEGs, 4.3%) was the highest representative terms for the VAT versus TAT comparison, followed by “Cytokine-cytokine receptor interaction” (34 DEGs, 3.2%) and “PI3K-Akt signaling pathway” (33 DEGs, 3.1%) (Table S6). “HTLV-I infection” (31 DEGs, 2.9%), “Rap1 signaling pathway” (27 DEGs, 2.6%), “Pathways in cancer” (27 DEGs, 2.6%), and “Chemokine signaling pathway” (26 DEGs, 2.5%) with relatively higher proportion of genes were the next most abundant pathways (Table S6), of these, “Cytokine-cytokine receptor interaction” was the first significantly highly enriched (*q* = 6.16E-03) pathway, with predominantly upregulated DEGs. When the subcutaneous fat was compared to visceral fat (SAT/VAT), similar pathways were found to be enriched but with less number of DEGs. Unexpectedly, upon SAT vs. TAT comparison, not even a single down-regulated gene was discovered in the significantly enriched pathways. For instance, CPE involved in human endocrine and metabolic diseases such as “Type I diabetes mellitus”, THBD involved in immune system such as “Complement and coagulation cascades”, and OSMR involved in signal transduction such as “Jak-STAT signaling pathway”, were evidently upregulated upon a slightly less stringent analysis.

### The clustering of DEGs among the three adipose depots

**Figure 10:**
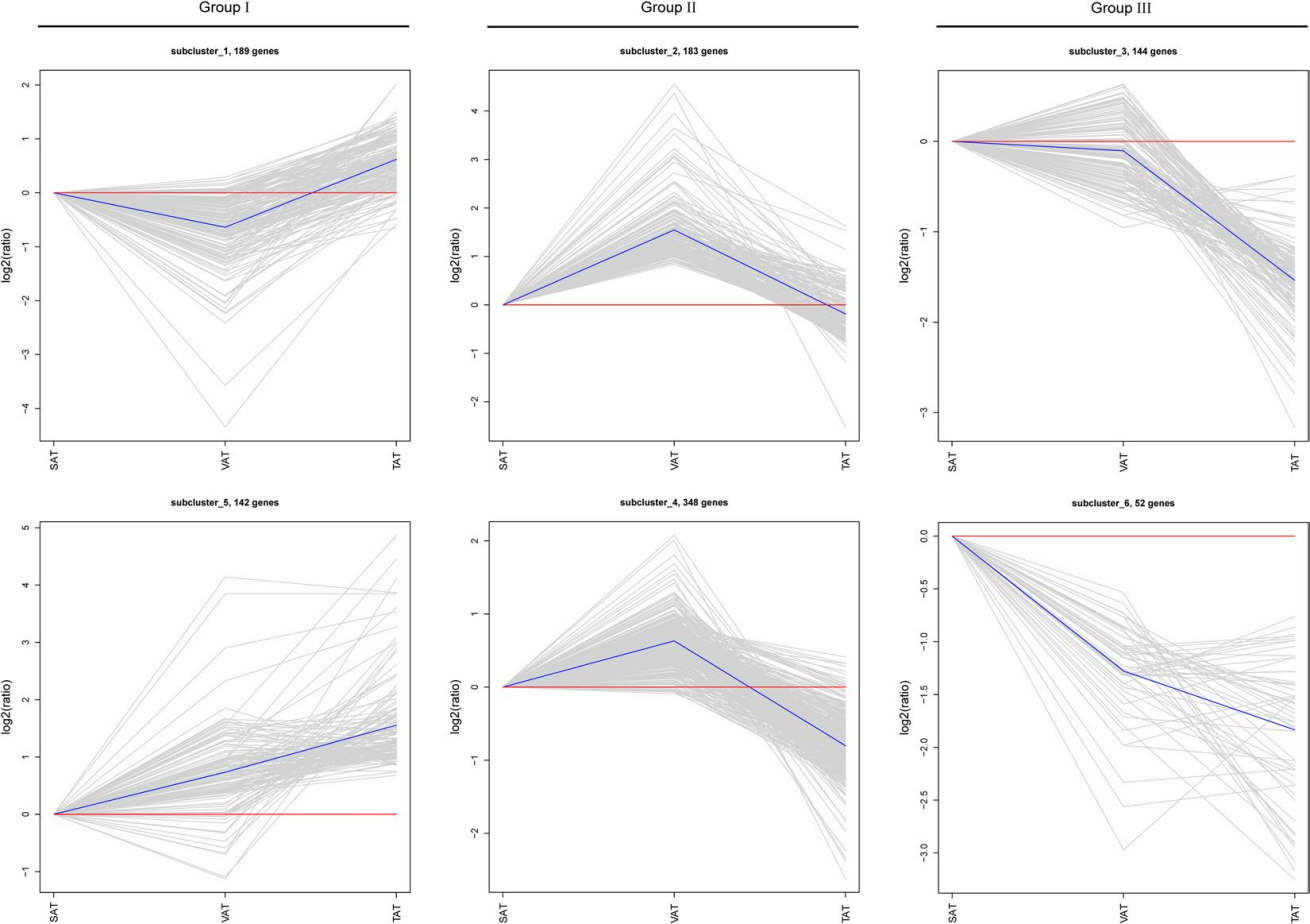
Hierarchical cluster analysis of DEGs from three pairs of comparisons. These genes can be clustered into six subclusters based on their expression patterns. Sub-clusters1–6: X-axis represents the different adipose depots (SAT, VAT and TAT); Y-axis indicates expression changes. The relative expression levels of genes in each subcluster are depicted by a gray line, the averaged expressions of all genes in the clusters are shown by a blue line. The numbers of DEGs in each subcluster are displayed at the top of plots.

To determine the primary patterns of gene expression, we further employed hierarchical clustering analysis of all DEGs based on their similar expression modulations. The 1,058 DEGs were partitioned into six different subclusters. According to the overall expression trend across the three adipose depots, the DEGs could be further divided into three groups including two up-regulated patterns (subcluster1 and subcluster5), two down-regulated patterns (subcluster3 and subcluster6), and two up/down patterns (subcluster2 and subcluster4). These subclusters consisted of 142, 144, 52, 189, 183, and 348 DEGs, respectively (Table S7, Fig. 10). We observed that the expression of genes from Group I were high in TAT but low in SAT and VAT, while the expression of genes from Group III were regularly decreased in TAT but increased in SAT and VAT (Table S7, Fig. 10). Several lipid-related GO categories including lipid metabolic process, lipid biosynthetic process, cellular lipid metabolic process and fatty acid derivative binding were enriched with 19, 10, 13 and 2 DEGs clustered in Group I, respectively (Fig. S6). These up-regulated genes in TAT were likely to play essential roles in the regulation of fat deposition in the tail region. For the Group II, the averaged expressions of genes reached peak values in VAT among three adipose types (Table S7, Fig. 10), which corresponded to more significantly enriched GO terms associated with inflammatory response, immune system and infectious diseases in Group II (Fig. S6). To ascertain the involvement of these DEGs in pathways, we further performed KEGG pathway analysis of DEGs in each subcluster and found that the genes in Group I were mainly from metabolic pathways (Table S8), such as FASN, PNPLA3 and BCO1, involved in “Fatty acid metabolism”, “Glycerolipid metabolism”, and “Retinol metabolism”, respectively. Genes in Group II were mostly related to immune and inflammation reaction, communication of signal molecules and disease processes (Table S8), such as the participation of JUN in “HTLV-I infection” and “T cell receptor signaling pathway”. In Group III, most of genes were also involved in the pathways of immune and infectious diseases (Table S8), for example, Class II MHC class antigens were participated in “Staphylococcus aureus infection”, indicating SAT may be an immune-privileged organ in certain physiological stages. As described in the previous study, metabolic, inflammatory and innate immune processes may be also coordinated and regulated by lipids^18^.

## Discussion

Adipose tissue is thought to be an energy-rich and heterogeneous endocrine organ, whose growth and development are determined by the adipocyte size and number. Different adipose types exhibit divergent adipocyte phenotypes, secretory functions and lipid metabolism based on their depots^2,19,20^, which may be essentially influenced by inherent genetic factors. In this study, we first analyzed the differences in adipocyte size and fatty acid composition in adipose tissues from different fat depots, then investigated global gene expression patterns by transcriptome profiling of three different adipose types in Tan sheep: subcutaneous, visceral, and tail using Illumina HiSeq 4000. We noted great changes between any two of the three adipose tissue transcriptomes. However, it needs to be highlighted that our data just represent gene expression trends of whole adipose tissues rather than a certain cell type or fraction, as there is a variety of different cell types in adipose tissue, including adipocytes, preadipocytes, fibroblasts, vascular endothelial cells, and immune cells.

We carried out a RNA-seq analysis of SAT/VAT, SAT/TAT and VAT/TAT and identified 1,058 DEGs, which highlights why the three adipose tissues differ in their biological activities. Among the three comparisons, the highest number of DEGs (795 in 1,058 DEGs, 75%) was identified in VAT vs. TAT, followed by SAT vs. TAT (324) and SAT vs. VAT (218). In PCA plot, TATs and VATs were separate as expected, which might be indicative of more specific lineage or functional characteristics between these two depots. To further survey the primary patterns of gene expression, we grouped the 1,058 DEGs by a hierarchical clustering analysis. We particularly focused on an extensive set of differentially expressed developmental genes. Homeobox gene HOXA10 differentially expressed between any two adipose types (Fig. 4), was clustered in Group I, which also included several HOX family members such as HOXC10, HOXC11, HOXC12, HOXC13, HOX-domain encoding genes such as IRX1, IRX3, and IRX5, and T-box gene TBX15 that were increased in TAT when compared with SAT or VAT (Table S7). A study of human adipose tissue depots have already reported that HOXC13 was expressed only in the gluteal depot and tended to have higher levels in females^21^. Some of these genes were significantly enriched in the first two categories, proximal/distal pattern formation and pattern specification process (Fig. S6). Previous studies demonstrated that HOX genes play key regulatory roles in anterior to posterior vertebrate axial morphology. Homeobox gene mutations or expression changes, or altered regulatory mode of downstream genes in a certain region would affect individual normal development in that area. To our knowledge, the caudal region differs markedly between short and fat-tailed sheep and is characterized by remarkable changes in fat mass, which might depend largely on the numbers of coccygeal vertebrae in vertebrates, this remains to be further investigated. Two opposite gain- and loss-of-function experiments on HOX genes in Drosophila and mouse further provided additional evidence that HOX genes promote their own identity programs, and any ectopic expression of HOX members could lead to anterior and posterior transformations, or increased or decreased vertebrae. Evidence from human and mouse studies indicated that developmental genes are present in fat tissues, even in undifferentiated precursor cells and mature brown fat cells, and are linked to lipid accumulation. The analysis for KEGG pathway indicated the involvement of HOXA9 and HOXA10 in the “Transcriptional misregulation in cancer”, playing a possible role in cell differentiation resistance. Although numerous studies of human and rodent models have greatly enhanced our understanding of the differences between visceral and subcutaneous fat, it has been very hard to translate these discoveries from human and rodents when we investigated tail fat tissue in sheep owing to the lack of comparable tail fat depots. Our data could reflect actual changes in expression level of developmental genes in adipose depots for sheep, our results strongly suggest that the up-regulated HOX genes in TAT may have an active role in regulating tail adipose deposition and morphological diversity in sheep tails. These findings provide new insights into exploring the molecular mechanism of tail fat deposition.

We also detected several representative DEGs that were abundantly expressed in TAT. For example, FASN (1839.77 for VAT, 3212.45 for SAT and 3873.70 for TAT, respectively) is primarily responsible for de novo lipogenesis in mammals, catalyzing the conversion of malonyl CoA into palmitate^22^; C/EPBα (133.42 for VAT, 201.44 for SAT, 347.26 for TAT, respectively), as one of the master adipogenic regulators, promotes the differentiation of preadipocytes into mature adipocytes^23^; G0S2 (981.55 for VAT, 1041.54 for SAT, 3205.27 for TAT, respectively) (Table 3), regulates white adipose tissue browning by serving as an inhibitor of adipose triglyceride lipase and resisting fat catabolism^24^, implying that TAT has a strong ability to synthesize and store triglycerides and a weak capability for adipose tissue lipolysis. Indeed, we also found a strong positive correlation between the expression levels of these genes and the volume of lipid-laden adipocyte (VAT < SAT < TAT) in the three types of adipose tissues (Fig. 1a and b). These genes can be considered as candidate genes that are potentially associated with adipose deposition in fat-tailed sheep. Moreover, we gave a special emphasis on the two genes that are involved in fatty acid transport, possibly impacting on fatty acid composition within local adipose tissues, SLC27A6 is responsible for catalyzing the transfer of intercellular monocarboxylic acids; CPT1A mediates the membrane transport of long fatty acids.

**Table 3:** The selected DEGs that were clustered in the three groups.

**Group**	**Gene_Symbol**	**FPKM**			**Gene_Description**
		**SAT**	**VAT**	**TAT**	
**Group I**					
	FASN	3212.453	1839.771	3873.703	Fatty acid synthase
	CEBPα	201.442	133.416	347.259	CCAAT/enhancer binding protein (C/EBP), alpha
	CLDN15	321.803	340.151	783.489	Claudin 15
	SREBF1	94.887	56.989	130.733	Sterol regulatory element binding transcription factor 1
	PNPLA3	48.199	29.403	54.552	Patatin-like phospholipase domain containing 3
	RXRα	23.710	13.230	28.251	Retinoid X receptor, alpha
	LRP3	3.430	2.749	6.381	Low density lipoprotein receptor-related protein 3
	G0S2	1041.538	981.552	3205.267	G0/G1 switch 2
	S100A8	5.615	3.114	113.266	S100 calcium binding protein A8
	FRZB	12.266	5.081	97.274	Frizzled-related protein
	PLIN5	28.23107	57.02489	58.40647	Perilipin 5
	S100A9	6.989	4.343	33.568	S100 calcium binding protein A9
	S100G	3.334	3.603	25.493	S100 calcium binding protein G
	BMP5	1.271	2.565	11.343	Bone morphogenetic protein 5
Group II					
	JUN	261.267	1007.498	249.664	Jun proto-oncogene
	NR4A1	61.515	228.872	41.053	Nuclear receptor subfamily 4, group A, member 1
	SFRP2	26.297	76.848	29.218	Secreted frizzled-related protein 2
	PDGFRB	27.615	44.707	16.218	Platelet-derived growth factor receptor, beta polypeptide
	ALDH1A3	9.288	26.591	1.900	Aldehyde dehydrogenase 1 family, member A3
	TCF21	0.080	24.450	0.093	Transcription factor 21
	DLK1	1.388	18.739	0.061	Delta-like 1 homolog (Drosophila)
	KLF7	6.307	10.019	2.924	Kruppel-like factor 7 (ubiquitous)
	FZD6	6.358	8.852	2.327	Frizzled class receptor 6
	CPT1A	3.603	5.985	2.028	Carnitine O-palmitoyltransferase 1, liver isoform
	GATA3	1.360	5.526	2.007	GATA binding protein 3
	CYP2E1	0.000	2.023	0.000	Cytochrome P450, family 2, subfamily E, polypeptide 1
	PPARGC1α	0.557	0.619	0.063	Peroxisome proliferator-activated receptor gamma, coactivator 1 alpha
Group III					
	GPAM	217.656	86.435	119.259	Glycerol-3-phosphate acyltransferase, mitochondrial
	FMO3	186.617	91.079	61.892	Flavin containing monooxygenase 3
	ABCA1	23.785	12.244	4.343	ATP-binding cassette, sub-family A (ABC1), member 1
	DKK3	11.573	14.683	2.165	Dickkopf WNT signaling pathway inhibitor 3
	BMP7	10.633	9.715	1.078	Bone morphogenetic protein 7
	WNT2	5.934	5.574	1.356	Wingless-type MMTV integration site family member 2
	AR	5.714	1.709	2.023	Androgen receptor

Aldehyde dehydrogenase 1 family member A3 (ALDH1A3) differentially expressed between any two of three adipose types (Fig. 4 and Fig.S4), was clustered in Group II, in which VAT had the highest mRNA expression levels (Table 3). The isoform ALDH1A3 encodes a member of the aldehyde dehydrogenase 1 family catalyzing the conversion of retinaldehyde to retinoic acid, a metabolite of Vitamin A, thus determining retinoic acid concentrations in adipocytes^25^. Retinoic acid stimulus in turn represses two central adipogenic transcription factors C/EBPα and C/EBPβ through a retinoic acid receptor-dependent mechanism that impedes the expression of PPARγ and resists lipid accumulation^26,27^. Our data showed that ALDH1A3 mRNA expression was increased in VAT relative to SAT and TAT (Table 3). These results revealed that ALDH1A3 might have a large impact on regional fat deposition. Such effect of ALDH1A1 (as one of the vitamin A-metabolizing enzymes) has already been confirmed in human visceral obesity^28^. Several key transcriptional regulators including GATA3, NR4A1 and KLF7 were found to be associated with adipocyte differentiation^29–31^; PPARGC1A has been found to play significant roles in brown fat adipogenesis and thermogenesis^32^; CPT1A is involved in fatty acid synthesis and degradation^33^; the EGF-like protein DLK1 is able to enhance adipogenic response of mesenchymal C3H10T1/2 cells^34^; Cytochrome P-450 CYP2E1 is expressed only in VAT, participating in the oxidation of several endogenous saturated and unsaturated fatty acids^35^; JUN was found to be participated in multiple inflammation and immune-associated pathways, which might reflect an increased requirement for protective immunity in the visceral fat depot. Overexpression of JUN could block adipogenesis by disturbing the C/EBPβ pathway^36^. Our data raise the possibility for significant roles played by these up-regulated genes in the development and maintenance of visceral adipose tissue.

Furthermore, two candidate regions located on chromosomes 5 and X, associated with fat deposition in the fat tail sheep have been identified by Moradi *et al*. (2012)^12^. In our data, we discovered many overlapping genes in these two genomic locations. Five of these genes, VSIG4, CLCN5, WAS, TIMP1 and AR, exhibited marked changes in expression among the three adipose depots. Tissue inhibitor of metalloproteinase 1 (TIMP-1) is an adipocyte-derived protein and its expression was induced by proinflammatory adipocytokine TNFα and IL-6^37^. The TIMP-1 over-expressed 3T3-L1 cells presented with accelerated lipid accumulation while the TIMP-1-deficient-mice could resist nutritionally induced obesity^37^. Hormone receptor density differences in the adipose tissues also influence regional fat distribution, for example, androgen receptor AR contributes to the control of adipocyte development by interacting with its own ligand androgen^38^. In addition, in a recent study, researchers identified eight important candidate genes, HOXA11, BMP2, PPP1CC, SP3, SP9, WDR92, PROKR1 and ETAA1, using genomic approaches^13^. These genes showed very low or undetectable expressions in our adipose samples except SP9 transcription factor that was uniquely expressed in TAT (Table S2). We deduced that SP9 is likely to participate in the process of Tan Sheep tail fat development by triggering commitment of mesenchymal stem cells to an adipocyte lineage, which need to be further studied.

In the network and pathway analysis, we identified some canonical pathways that have been documented to be at work during adipogenesis and/or lipid metabolic process, such as “Fatty acid metabolism”, “Wnt signaling pathway”, “TGF-beta signaling pathway”, “Hedgehog signaling pathway”, “ECM-receptor interaction”, and “PPAR signaling pathway”. In our results, despite no significant enrichment, we observed that several DEGs pivotal in fat formation were involved in these pathways. For example, FASN that participates in “fatty acid biosynthesis”, exhibits apparent up-regulation, while CPT1A that participates in “fatty acid degradation”, is notably down-regulated in the tail fat when compared to visceral fat (Table 3). These observations support the idea that there are more stored triglycerides in tail adipose tissue. We also found several Wnt-related genes, such as DKK3, FRZB and SFRP2, which act as antagonists and inhibitors of Wnt signals; WNT2 and FZD6, known as Wnt-ligand and transmembrane Fz-receptor respectively, constitute Wnt-Fz signaling to prevent adipogenesis, and have been widely described for their participation in the accumulation of fat mass^39^. Besides, a few members of the TGF-β pathway, including PITX2, NOG, BAMBI, BMP5 and BMP7 (Table 4), display differences in regulating regional deposition of adipose tissue. Low expression of BMP7 in tail adipose tissue and a high tail fat mass support the fact that BMP7 has the potential to accelerate osteogenesis^40^ and energy expenditure by activating brown adipocytes^41^. These DEGs cooperate with each other to exercise their biological functions.

**Table 4:** Differentially expressed genes that were involved in the signal transduction event associated with adipogenesis.

**Pathway**	**Gene_Symbol**	**Gene_Name**	**FPKM**			**log**_2_ **FoldChange**			**FDR**		
			**SAT**	**VAT**	**TAT**	**SAT/VAT**	**SAT/TAT**	**VAT/TAT**	**SAT/VAT**	**SAT/TAT**	**VAT/TAT**
Wnt signaling pathway											
	SFRP2	Secreted frizzled-related protein 2	26.297	76.848	29.218	1.437	—	—	0.007	—	—
	LOC101102299	Protein Wnt-10b	0.231	0.973	0.048	2.051	—	4.170	0.013	—	0.000
	JUN	Jun proto-oncogene	261.267	1007.498	249.664	1.849	—	1.671	0.012	—	0.040
	FZD6	Frizzled class receptor 6	6.358	8.852	2.327	—	—	1.605	—	—	0.006
	BAMBI	BMP and activin membrane-bound inhibitor homolog precursor	1.834	5.840	1.283	—	—	1.937	—	—	0.045
	PRKCB	Protein kinase C, beta	1.044	1.979	0.226	—	—	2.865	—	—	0.000
	WNT2	Wingless-type MMTV integration site family member 2	5.934	5.574	1.356	—	—	1.882	—	—	0.021
TGF-beta signaling pathway											
	PITX2	Paired-like homeodomain 2	0.621	0.053	0.022	3.536	4.776	—	0.040	0.008	—
	BMP5	Bone morphogenetic protein 5	1.271	2.565	11.343	—	3.489	2.473	—	0.000	0.000
	NOG	Noggin	1.107	1.811	5.390	—	2.487	1.846	—	0.001	0.010
	BAMBI	BMP and activin membrane-bound inhibitor homolog precursor	1.834	5.840	1.283	—	—	1.937	—	—	0.045
	BMP7	Bone morphogenetic protein 7	10.633	9.715	1.078	—	—	3.011	—	—	0.000
Notch signaling pathway											
	DTX4	Deltex 4, E3 ubiquitin ligase	9.344	2.573	8.210	1.952	—	1.988	0.001	—	0.000
	HES1	Hes family bHLH transcription factor 1	19.663	59.077	21.734	1.501	—	—	0.006	—	—
	NOTCH2	Notch 2	21.990	9.713	9.473	1.232	—	—	0.045	—	—
Hedgehog signaling pathway											
	PTCH2	Patched 2	49.568	31.503	76.816	—	—	1.571	—	—	0.002
	GLI3	GLI family zinc finger 3	1.281	1.999	0.500	—	—	1.751	—	—	0.008

Three sub pathways, “Cytokine-cytokine receptor interaction”, “Cell adhesion molecules”, and “ECM-receptor interaction”, are classified as “Signaling molecules and interaction”. These pathways interact and are dependent on each other. Extracellular matrix (ECM)-receptor interaction is known to influence expansion of fat mass and depot-specific adipogenesis^9^. Its structural components are mainly composed of collagens, elastins, proteoglycans, glycoproteins and some transmembrane receptors such as integrins which act as a bridge between the ECM and cells^9^. In this study, we found that three integrin genes (α1, α4, β7) to be principally expressed in VAT (Table S9). The ECM constituents, TNXB (tenascin XB), COL1A1 (collagen, type I, alpha 1) and COL11A2 (collagen, type XI, alpha 2) were found to be predominantly expressed in TAT (Table S9). It is worth mentioning here that cytokines and hormones in adipose tissue partially contribute to depot specific fat formation. Adipose tissue mainly contains adipocytes, vascular endothelial cells, immune cells, connective tissue matrix and nerve tissue^1^. These components contribute to adipose tissue function by interacting with each other. From our enriched KEGG pathways, more valuable information on the communication between cell types and within cells is available, especially for adipocytes and immune cells. For example, TNF-α, a known cytokine family member mainly secreted by immune cells, is able to inhibit adipocyte differentiation by down regulating key adipogenic genes such as C/EBPα, PPARγ and adiponectin. Certain white adipocyte-derived adipokines appear to contribute dramatically to immune and chronic inflammatory responses^42^. One of the TNF-α transmembrane receptors TNFRSF1B showed relatively higher expression level in VAT than in SAT or TAT (Table S9). The previous study has characterized the negative PDGF effect on 3T3-L1 and human preadipocyte differentiation through PKC-dependent signaling pathways, accompanied by a reduction of PDGF receptor^43^. In our data, the receptor PDGFRβ was markedly down-regulated in TAT (Table S9). This indicates that the adipogenic gene expression profile may be affected by PKC inhibition. Most of the other DEGs participate in the “Cytokine-cytokine receptor interaction” pathway, encoding cytokines and their corresponding receptors expressed at higher abundance in the visceral fat than the other two subcutaneous depots (Table S9). These observations are in agreement with the previous publication and provide further evidence that the visceral fat is more likely to elicit inflammatory response than the other two subcutaneous compartments^3^. Perhaps these cytokines affect adipose tissue microenvironment partially, by receptor-mediated intercellular signal transmission to alter cell type profile, resulting in a population of cells that changed to a reverse promoted inflammatory adipocyte phenotype^44^.

Another significantly enriched pathway is “Cell adhesion molecules” (CAMs) that mediates cell-cell contacts and the interactions between cells and extracellular matrix. Few of the CAMs on the surface of fat cells have been speculated to be of great importance to immune cell-adipocyte “crosstalk”, including ACAM, NCAM and ICAM-1^44^. A study focusing on tight junction proteins suggested that CLDN6 facilitates 3T3-L1 adipocyte maturation^45^. However, it was not detected in our study, but another CLDN family member (i.e. CLDN15) with unknown function in adipose tissue, was abundantly expressed in the three depots (Table S9), suggesting that CLDN15 might have a similar regulatory effect on fat deposition as CLDN6. Taken together, the majority of DEGs involved in this pathway were strikingly increased in VAT when compared to TAT or SAT (Table S9).

We also identified a few DEGs that were enriched in endocrine system. For instance, RXRα, DBI, and SLC27A6 were enriched in PPARs signaling pathway (Table 5) and are known to chiefly regulate adipocyte differentiation. In the insulin-related pathways and adipocytokine signaling pathway, the enriched DEGs were mainly from the VAT vs. TAT comparison (Table 5), which further showed great differences in the visceral fat and tail fat.

**Table 5:** Differentially expressed genes that were involved in the immune system and endocrine system.

**Pathway**	**Gene_Symbol**	**Gene_Name**	**FPKM**			**log**_2_ **FoldChange**			**FDR**		
			**SAT**	**VAT**	**TAT**	**SAT/VAT**	**SAT/TAT**	**VAT/TAT**	**SAT/VAT**	**SAT/TAT**	**VAT/TAT**
PPAR signaling pathway											
	RXRA	Retinoid X receptor, alpha	23.710	13.230	28.251	—	—	1.381	—	—	0.013
	DBI	Diazepam binding inhibitor (GABA receptor modulator, acyl-CoA binding protein)	1294.623	808.037	1827.389	—	—	1.471	—	—	0.006
	SLC27A6	Solute carrier family 27 (fatty acid transporter), member 6	3.832	1.117	2.073	1.814	—	—	0.013	—	—
Adipocytokine signaling pathway											
	PPARGC1A	Peroxisome proliferator-activated receptor gamma, coactivator 1 alpha	0.557	0.619	0.063	—	2.929	—	—	0.033	—
	AKT2	V-akt murine thymoma viral oncogene homolog 2	111.075	76.556	142.164	—	—	1.200	—	—	0.043
	TNFRSF1B	Tumor necrosis factor receptor superfamily, member 1B	15.132	22.681	6.001	—	—	1.624	—	—	0.003
	CAMKK1	Calcium/calmodulin-dependent protein kinase kinase 1, alpha	9.824	4.345	14.618	—	—	2.050	—	—	0.000
Insulin signaling pathway											
	SREBF1	Sterol regulatory element binding transcription factor 1	94.887	56.989	130.733	—	—	1.523	—	—	0.028
	PIK3R5	Phosphoinositide-3-kinase, regulatory subunit 5	2.010	2.684	0.766	—	—	1.512	—	—	0.033
	SHC2	SHC (Src homology 2 domain containing) transforming protein 2	2.712	2.622	11.250	—	2.297	2.420	—	0.000	0.000
	CBL	Cbl proto-oncogene, E3 ubiquitin protein ligase	4.834	2.656	1.445	—	1.512	—	—	0.034	—
	CBLB	Cbl proto-oncogene B, E3 ubiquitin protein ligase	3.659	6.384	1.011	—	1.568	2.355	—	0.025	0.000
Insulin secretion											
	LOC105606031	Adenylate cyclase type 5-like	0.510	0.317	0.956	—	—	1.858	—	—	0.012
	KCNN3	Potassium channel, calcium activated intermediate/small conductance subfamily N alpha, member 3	1.638	3.977	0.576	—	—	2.469	—	—	0.000
	ADCYAP1R1	Adenylate cyclase activating polypeptide 1 (pituitary) receptor type I	2.570	0.428	0.467	2.653	—	—	0.035	—	—
	CREB5	cAMP responsive element binding protein 5	7.442	13.803	3.418	—	—	1.704	—	—	0.006
	CREB3L2	cAMP responsive element binding protein 3-like 2	9.003	8.142	2.493	—	1.597	1.429	—	0.019	0.032
	ADCY7	Adenylate cyclase 7	2.597	3.874	1.084	—	—	1.533	—	—	0.015
	ADCY5	Adenylate cyclase 5	8.749	8.025	22.870	—	1.603	1.778	—	0.008	0.000

In conclusion, we measured the adipose phenotype and sequenced the transcriptome of the three sources of adipose tissues in Tan Sheep using Illumina Hiseq 4000 sequencing platform. A total of 1,058 DEGs were identified between subcutaneous, visceral and tail fat tissues, with the significance level of *p* < 0.05. Based on the GO and KEGG enrichment results, we discovered some differentially expressed genes that were potentially associated with regional fat distribution and tail adipose tissue enlargement. More importantly, our results indicated that HOX and HOX-related genes might play novel potential roles in regulating regional fat distribution and diverse tail types in fat-tailed sheep breed. These findings provide a foundation for studying the regulation of fat deposition in sheep in the future, and also contribute to our understanding of undesirable adipose deposits in certain regions of the human body.

## Materials and Methods

### Ethical statement

All the animal procedures were carried out in accordance with the guidelines of the China Council on Animal Care and the Ministry of Agriculture of the People’s Republic of China. All animal experiments were approved by Review Committee for the Use of Animal Subjects of Northwest A&F University.

### Animal characteristics and tissue collection

The study was conducted on adult ewes (aged approximately 3.5 years) obtained from the Tan sheep herd in the Ningxia Tianyuan Tan Sheep Farm (Hongsibu, Ningxia). A total of three healthy fat-tailed individuals were selected randomly. The three types of adipose tissues i.e., subcutaneous, visceral and tail were taken from the thoracic, perirenal and tail regions, respectively. Each fat tissue was divided into three parts: the first two parts were separately used for staining and determining fatty acid composition while the third part was immediately submerged in liquid nitrogen, and stored at −80 °C for RNA-seq analysis.

### Adipose measurements

Fat samples fixed in 4% paraformaldehyde solution were embedded in paraffin, cut into 6 μm sections using RM2235 rotary microtome (Leica, Germany) and stained with hematoxylin and eosin (H&E). Cell morphology and adipocyte size was noted for each sample. More specifically, we cut three slices per tissue, randomly selected five visual fields (×200) in each section to collect images under a microscope imaging and analysis system (Nikon Ds-Ri2, Japan), measured the maximum and minimum diameters of 100 fat cells, and calculated the geometric average as the average diameter. The mean fat cell volume was obtained by the following formula^46,47^:

$${\rm{V}}=\pi /6(3{s}^{2}\bar{d}+{\bar{d}}^{3})$$

where $\overline{d}$ is the average diameter of the fat cells and *s* is the standard deviation. The fatty acid composition of three adipose depots were determined in triplicate. Briefly, total lipids were extracted using a previously described method^48^ with one minor modification, methyl esterified with n-hexane and analyzed on a gas chromatograph (Agilent 7820a, USA) equipped with a flame ionization detector and capillary column (HP-88, length 100 m, inner diameter 0.25 mm, film thickness 0.20 μm, USA). Data were analyzed by One-way ANOVA with Tukey multiple range test (n = 3) of *p* value below 0.05. To further determined the differences between any two of three adipose types, pairwise multiple comparison was performed, and *p*1, *p*2 and *p*3 represented SAT vs VAT, SAT vs TAT and VAT vs TAT, respectively. Values were presented as means ± SE.

### RNA isolation and quality evaluation

TRIzol reagent (Invitrogen, USA) was used to extract total RNA from 9 tissue samples according to the manufacturer’s recommended protocol. Subsequently, extracts were fractionated on 1% agarose gels to monitor RNA degradation and contamination, followed by assessment of RNA yield with Qubit® RNA Assay Kit in Qubit® 2.0 Flurometer (Life Technologies, CA, USA). Purity and integrity of RNA was checked with the NanoPhotometer® spectrophotometer (IMPLEN, CA, USA) and RNA Nano 6000 Assay Kit of the Bioanalyzer 2100 system (Agilent Technologies, CA, USA) with RIN number > 6.8.

### Library construction and transcriptome sequencing

Poly(A) mRNA isolated from approximately 3 μg of total RNA with poly-T oligo-attached magnetic beads (Invitrogen) was cleaved into shorter fragments using a metal catalyst under elevated temperature. The cleaved RNA fragments were reverse-transcribed to generate first-strand cDNA, accompanied with second-strand cDNA synthesis using Invitrogen DNA Polymerase I and RNase H (New England Biolabs). The short cDNA fragments generated were purified, end-repaired, poly-adenylated and ligated to indexed sequencing adaptors. To selectively enrich library fragments ranging from 150 to 200 bp in length, the ligated cDNA molecules were purified with AMPure XP system (Beckman Coulter, Beverly, USA), and used as templates for PCR amplification based on the results of agarose gel electrophoresis. After gel purification, the amplified PCR products were subjected to quality assessment on the Agilent Bioanalyzer 2100 system. Eventually, the paired-end reads (150 bp) of the libraries were sequenced on the Illumina Hiseq 4000 platform (LC Sciences, USA).

### Alignment of reads and analysis of differential gene expression

Raw reads from each sequencing library were first processed to discard adaptor sequences, unknown sequences and low quality sequences. Meanwhile, data filtering including Q20, Q30, GC content and sequence duplication level were conducted based on the quality parameters of the retained clean reads. *Ovis aries* reference genome sequence and the corresponding annotated transcript set were directly downloaded from the sheep genome website (ftp://ftp.ensembl.org/pub/release-82/fasta/ovis_aries/dna/). TopHat v2.0.12 was employed to align the high quality clean reads to the reference sequence creating an index using Bowtie v2.2.3 with default parameters. The number of paired-end reads mapped to each gene were calculated and normalized to fragments per kilobase of exon per million mapped reads (FPKM). Differential gene expression between any two fat depots was estimated with DESeq package in R (1.18.0). Ratios of SAT/VAT, SAT/TAT, and VAT/TAT were used to compare fold-changes among pairs of adipose types. In our analysis, a corrected *p*-value (false discovery rate, FDR) ≤ 0.05 and an absolute value of log_2_ (fold change) ≥ 1 were set as thresholds for differential expression^49^. Further expression pattern analysis was conducted on all obtained DEGs.

### Quantitative real-time PCR validation

Nine selected genes including FRZB, SFRP2, NR4A1, CLDN15, SCD1, PDGFD, DLK1, TCF21 and RDH16, and fourteen differentially expressed HOX genes including HOXA6, HOXA9, HOXA10, HOXB2, HOXB5, HOXB7, HOXC4, HOXC6, HOXC8, HOXC9, HOXC10, HOXC11, HOXC12 and HOXC13, identified by RNA-seq approach, were validated using q-PCR. Primers spanning exon-exon boundaries were designed to enhance the specificity of the q-PCR reaction (Table S10). Actin beta was used as a reference control gene to normalize mRNA expression. The q-PCR reaction was run on a CFX Connect Real-Time PCR Detection System (Bio-Rad) using SYBR Premix Ex Taq (TaKaRa). The reaction conditions were as follows: denaturation at 95 °C for 10 min, followed by 40 cycles of amplification (95 °C for 15 s, 60 °C for 30 s, and 72 °C for 30 s). The 2^−△△Ct^ method was used for calculating the relative abundance of genes^50^. All q-PCR experiments had an average technical replicate value for each of the three biological replicates.

### GO category and KEGG pathway enrichment of DEGs

Gene Ontology (GO) based on hypergeometric test is an international standard gene functional classification system^51^. In this work, all DEGs were mapped to terms in the GO database with the GOseq R package. An FDR-corrected *p* value of less than 0.05 was designated as the threshold to determine significantly enriched GO terms in DEGs when compared with the genomic background^52^.

Kyoto Encyclopedia of Genes and Genomes (KEGG, http://www.genome.jp/kegg) is an integrated reference database resources for biological interpretation of genome sequences and other high-throughput data^53^. We selected KOBAS software to find the statistically enriched DEGs in KEGG pathways based on a given *p* value (*p* ≤ 0.05).

### Novel transcript predictions

Cufflinks v2.1.1 Reference Annotation Based Transcript (RABT) assembly method was used to construct and identify both known and novel transcripts from TopHat alignment results.

### Data Availability

All the basic data series were submitted to NCBI’s Sequence Read Archive with accession number SRP094676.

## Electronic supplementary material

Supplementary information

Dataset 1

Dataset 2

Dataset 3

Dataset 4

Dataset 5

Dataset 6

Dataset 7

Dataset 8

Dataset 9

Dataset 10
