# Supplementary material for: Comparative transcriptome analysis reveals potentially novel roles of Homeobox genes in adipose deposition in fat-tailed sheep
Source: Sci Rep. 2017 Nov 3;7:14491. doi: 10.1038/s41598-017-14967-9 (PMC5670210; doi:10.1038/s41598-017-14967-9)
Supplement: Supplementary file 1 — Supplementary information [file 41598_2017_14967_MOESM1_ESM.pdf]

Comparative transcriptome analysis reveals potentially novel roles of Homeobox genes in adipose deposition in fat-tailed sheep

Danju Kang, Guangxian Zhou, Shiwei Zhou, Jie Zeng, Xiaolong Wang, Yu Jiang, Yuxin Yang*, and Yulin Chen*

College of Animal Science and Technology, Northwest A&F University, No. 22, Xinong Road, Yangling 712100, Shaanxi, China

*Correspondence: chenyulin@nwafu.edu.cn (Y.C), yangyuxin2002@126.com (Y.Y)

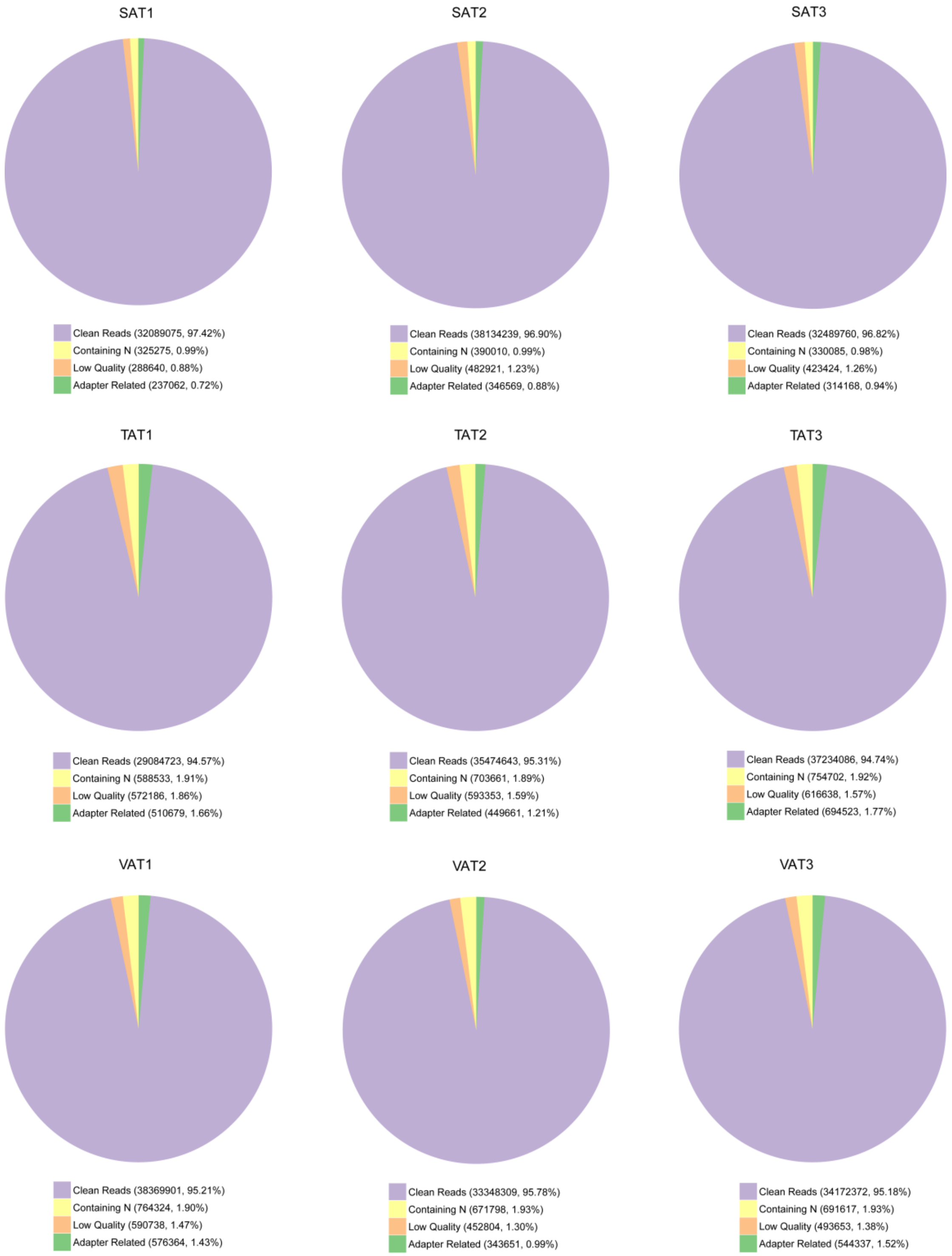

Supplementary Fig. S1. Classification of raw reads from nine adipose libraries. Raw reads contain clean reads, containing N, low quality and adapter related reads. Panels are orderly for SAT1, SAT2, SAT3, TAT1, TAT2, TAT3, VAT1, VAT2, and VAT3, respectively.

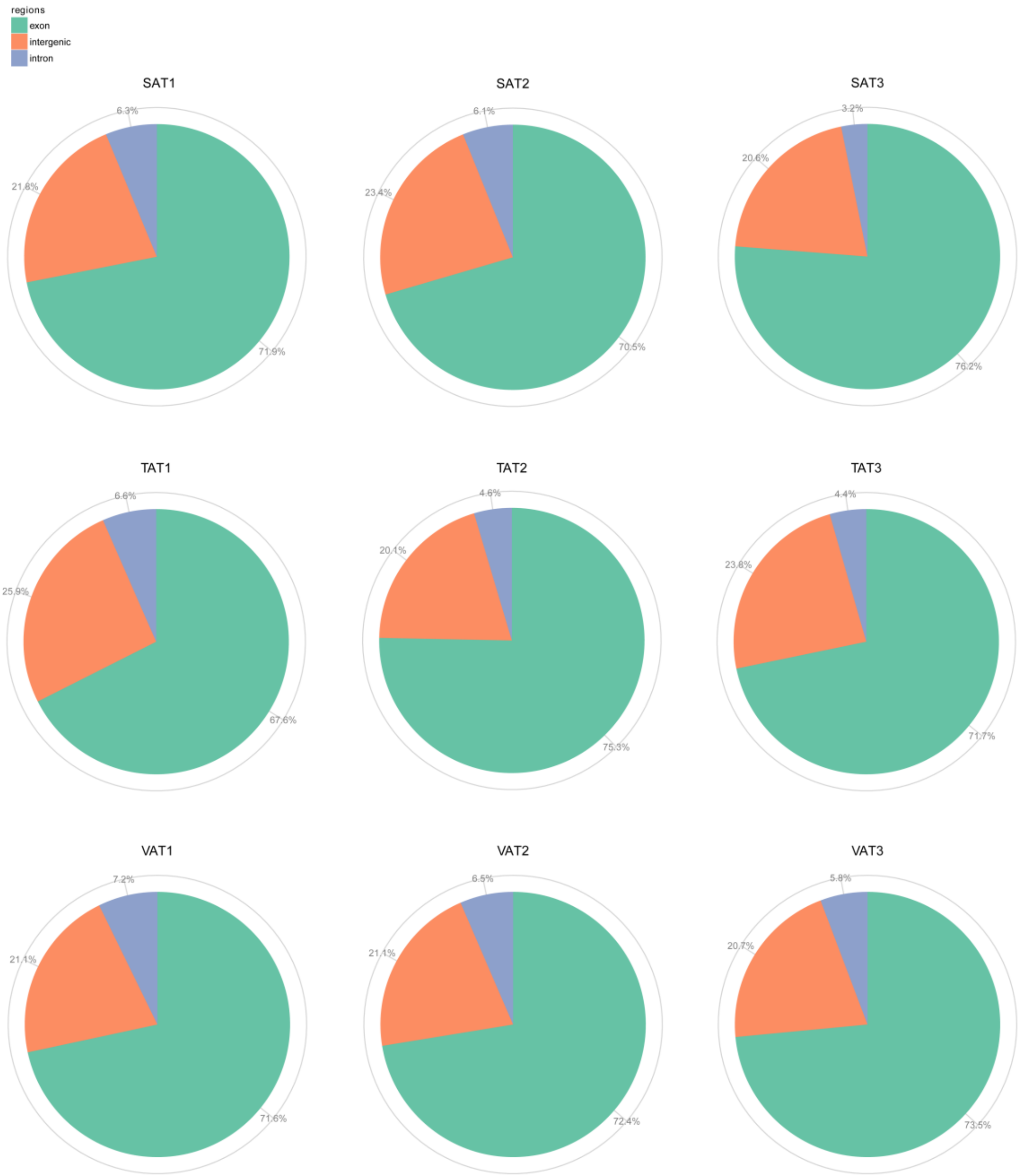

Supplementary Fig. S2. Distribution of clean reads. Clean reads were mapped to different regions of the reference genome, including exon, intergenic and intron locations. Panels are orderly for SAT1, SAT2, SAT3, TAT1, TAT2, TAT3, VAT1, VAT2, and VAT3, respectively.

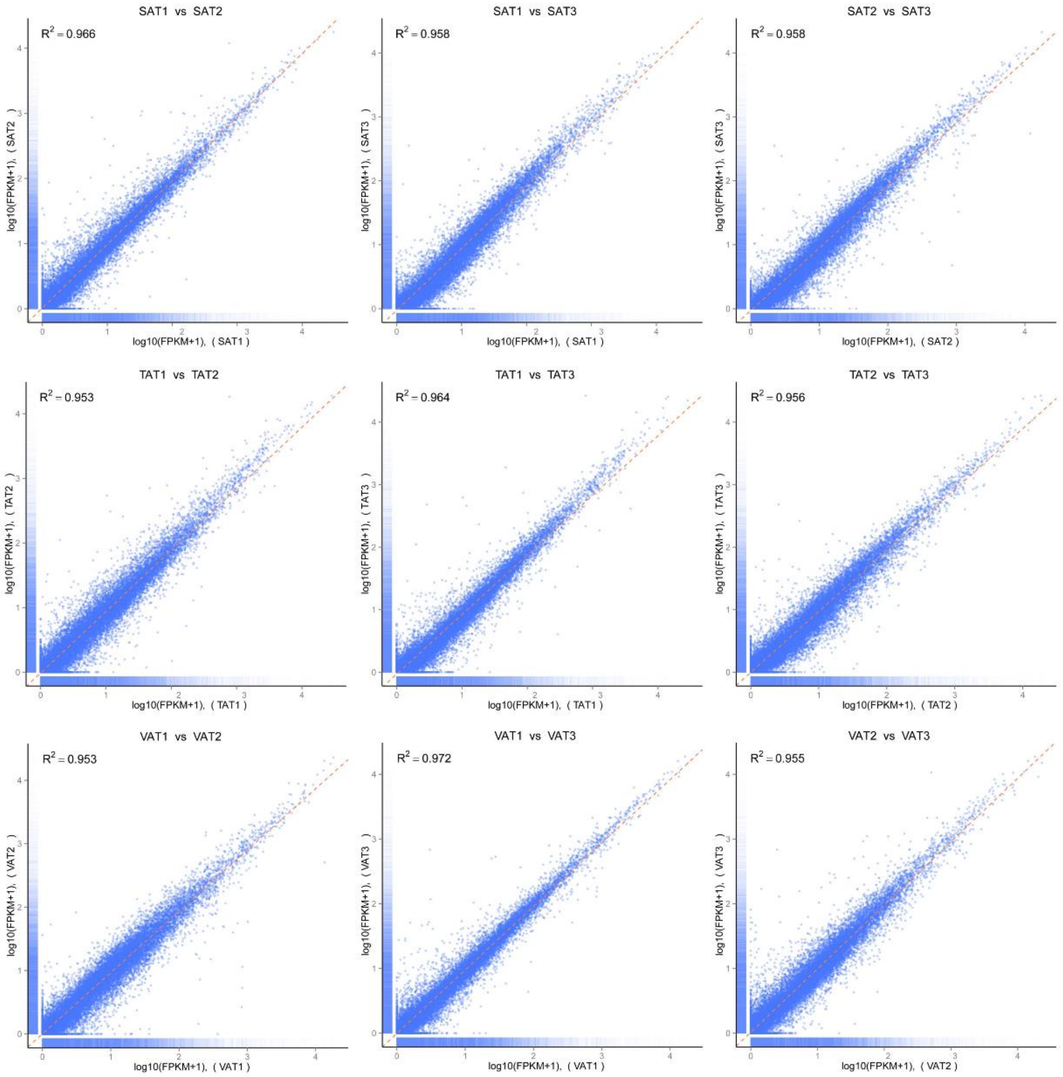

Supplementary Fig. S3. Distribution of the pair-wise correlation (R) between any two biological replicates under the same condition (same type of adipose). X and Y-axis represent the normalized FPKM values of any two biological replicates. There are three biological replicates for each adipose depot in this study, therefore generating three pairs of adipose comparisons for each depot.

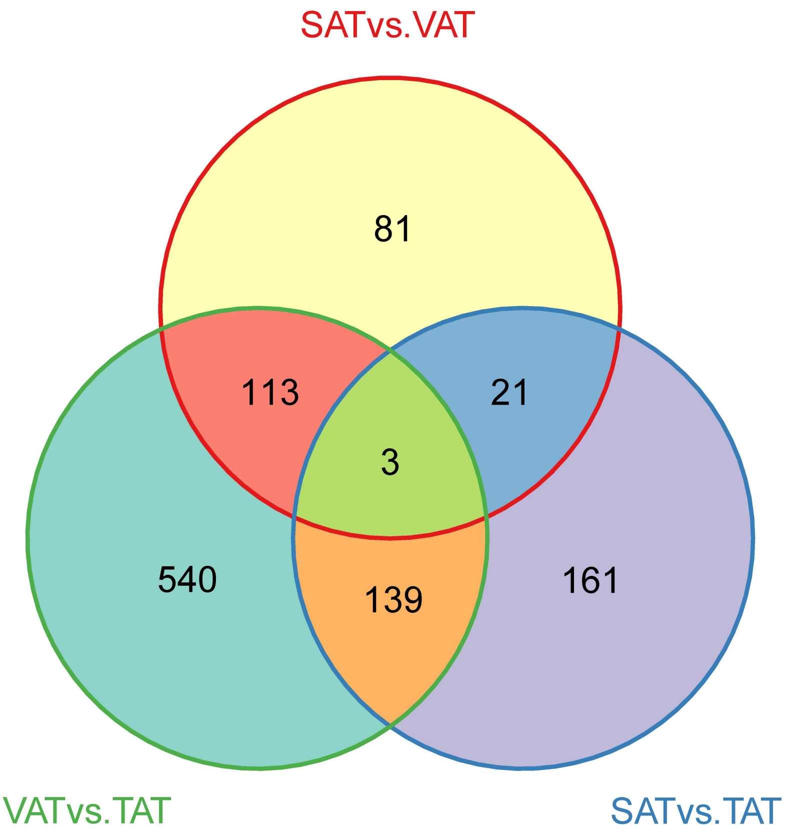

Supplementary Fig. S4. Unique and co-expressed genes in SAT vs. VAT, SAT vs. TAT, and VAT vs. TAT. Each comparison is represented by a circle in the Venn diagram. All 1,058 DEGs are composed of seven parts, of which the four overlapping regions indicate the shared genes in two or three comparisons while the three non-overlapping domains indicate the uniquely expressed genes among pairs of depots. Only three genes were co-expressed in all three comparisons. VAT vs. TAT had the highest number (540) of unique genes, and it shared 139 genes with SAT vs. TAT and 113 genes with SAT vs. VAT. SAT vs. VAT and SAT vs. TAT co-expressed 21 genes, while each comparison was represented by 81 and 161 unique genes, respectively.

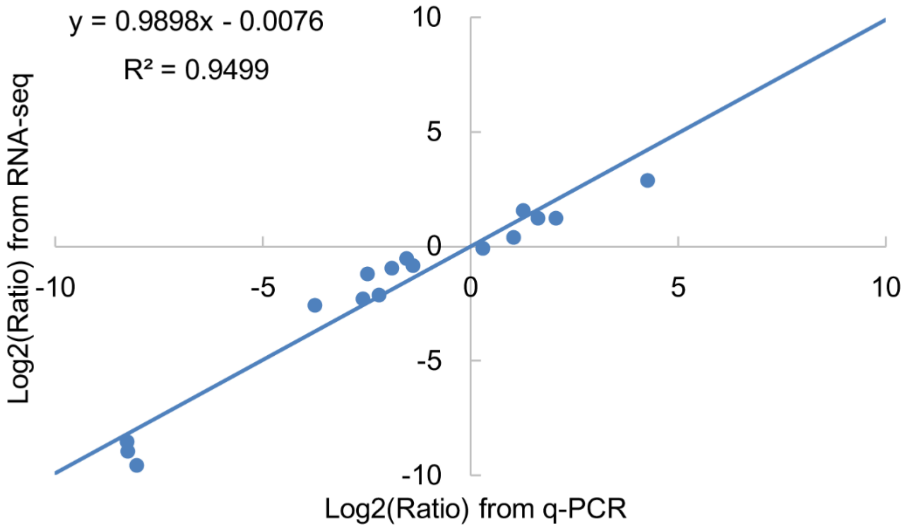

Supplementary Fig. S5. Coefficient analysis of fold change data between q-PCR and RNA-seq. Nine genes were selected for q-PCR experiment. Scatter diagrams were generated by the log2 expression ratios from RNA-seq (X-axis) and q-PCR (Y-axis). R-squared value was up to 0.95.

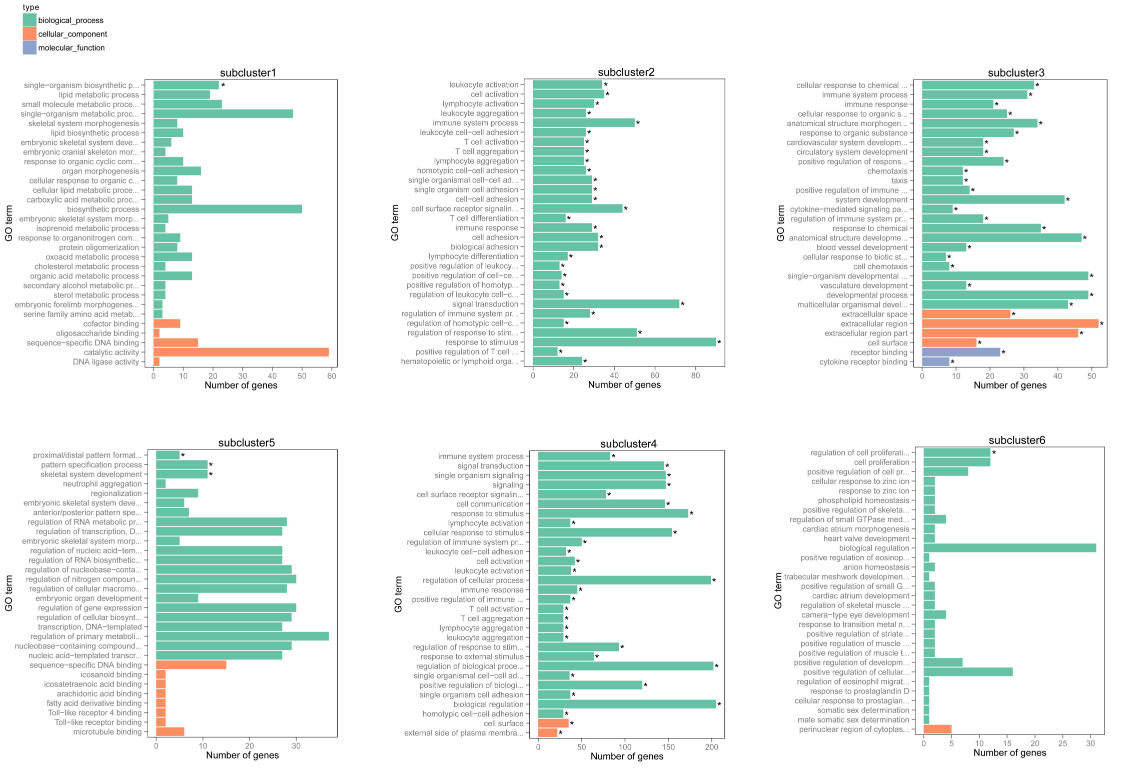

Supplementary Fig. S6. The most enriched GO terms in each subclusters. X-axis represents number of genes that were enriched in each term, y-axis represents the top 30 GO terms based on the corrected *p* value. * indicates the corrected $p < 0.05$. Biological process, cellular component and molecular function are denoted with green, orange and gray, respectively.

Supplementary Table S1. Statistics for gene numbers and percentage within different gene expression level intervals.

Supplementary Table S2. Genes that differentially expressed in the comparisons of SAT vs. VAT, SAT vs. TAT and VAT vs. TAT.

Supplementary Table S3. Differences in chromosomal location and number of HOX genes among five species of mammals.

Supplementary Table S4. Biological progress GO terms of DEGs from three comparison groups among different adipose depots.

Supplementary Table S5. Cellular components and molecular function GO terms of DEGs from three comparison groups among different adipose depots.

Supplementary Table S6. List of KEGG pathways for DEGs between any two of three adipose types.

Supplementary Table S7. List of genes with different expressions in six subclusters.

Supplementary Table S8. The percentage of the DEGs from six subclusters in 237 KEGG pathways.

Supplementary Table S9. Differentially expressed genes that were involved in the signaling molecules and interaction pathways.

Supplementary Table S10. Primer sequences used for q-PCR validation of twenty-four DEGs.
